# Adenoviral TMBIM6 vector attenuates ER-stress-induced apoptosis in a neonatal hypoxic-ischemic rat model

**DOI:** 10.1242/dmm.040352

**Published:** 2019-11-22

**Authors:** Desislava Doycheva, Ningbo Xu, Harpreet Kaur, Jay Malaguit, Devin William McBride, Jiping Tang, John H. Zhang

**Affiliations:** 1 Center for Neuroscience Research, Department of Physiology and Pharmacology, Basic Sciences, Loma Linda University School of Medicine, Loma Linda, CA 92354, USA; 2 Department of Interventional Radiology, Zhujiang Hospital, Southern Medical University, Guangzhou 510282, China; 3 The Vivian L. Smith Department of Neurosurgery, McGovern Medical School, The University of Texas Health Science Center at Houston, Houston, TX 77030, USA; 4 Departments of Anesthesiology, Neurosurgery and Neurology, Loma Linda University School of Medicine, Loma Linda, CA 92354, USA

**Keywords:** Neonatal hypoxia ischemia, Bax inhibitor 1, TMBIM6, ER stress, Apoptosis

## Abstract

Endoplasmic reticulum (ER) stress is a major pathology encountered after hypoxic-ischemic (HI) injury. Accumulation of unfolded proteins triggers the unfolded protein response (UPR), resulting in the activation of pro-apoptotic cascades that lead to cell death. Here, we identified Bax inhibitor 1 (BI-1), an evolutionarily conserved protein encoded by the transmembrane BAX inhibitor motif-containing 6 (*TMBIM6*) gene, as a novel modulator of ER-stress-induced apoptosis after HI brain injury in a neonatal rat pup. The main objective of our study was to overexpress BI-1, via viral-mediated gene delivery of human adenoviral-TMBIM6 (Ad-TMBIM6) vector, to investigate its anti-apoptotic effects as well as to elucidate its signaling pathways in an *in vivo* neonatal HI rat model and *in vitro* oxygen-glucose deprivation (OGD) model. Ten-day-old unsexed Sprague Dawley rat pups underwent right common carotid artery ligation followed by 1.5 h of hypoxia. Rat pups injected with Ad-TMBIM6 vector, 48 h pre-HI, showed a reduction in relative infarcted area size, attenuated neuronal degeneration and improved long-term neurological outcomes. Furthermore, silencing of BI-1 or further activating the IRE1α branch of the UPR, using a CRISPR activation plasmid, was shown to reverse the protective effects of BI-1. Based on our *in vivo* and *in vitro* data, the protective effects of BI-1 are mediated via inhibition of IRE1α signaling and in part via inhibition of the second stress sensor receptor, PERK. Overall, this study showed a novel role for BI-1 and ER stress in the pathophysiology of HI and could provide a basis for BI-1 as a potential therapeutic target.

## Abstract (precis)

**Summary:** Elucidating the role of Bax inhibitor 1 (BI-1) and its mechanisms in the pathophysiology of hypoxic ischemic encephalopathy may help provide a basis for BI-1 as a potential therapeutic target in clinics.

## INTRODUCTION

Hypoxic-ischemic brain injury, as a result of hypoxemia or reduced cerebral blood flow (Ferriero, 2004), causes pulmonary immaturity, respiratory distress syndrome, hypercapnia, hypoperfusion, seizures and long-term cognitive and behavioral deficits (Bracewell and Marlow, 2002; Ferriero, 2004; Yang et al., 2015; Wirrell et al., 2001; Bartha et al., 2007). Hypoxia ischemia (HI) affects 1–4 cases per 1000 births (Vannucci, 2000) with morbidity rates ranging from 10–80% based on the severity of injury (Chaudhari et al., 2014; Kaser et al., 2008). Current treatments include hypothermia, anticonvulsants, fluid and electrolytes management, and drugs such as atropine and epinephrine (Zanelli et al., 2011; Jacobs et al., 2013), but despite these treatments, 40–50% of treated patients still have persistent neurodevelopmental deficits (Zanelli et al., 2011; Barrett et al., 2007).

The endoplasmic reticulum (ER) is responsible for correct protein folding and protein function (Zhang et al., 2015). It is crucial for cell survival; however, under pathological conditions, such as HI, there is an accumulation of unfolded proteins that exceeds the capacity of the chaperones to fold the proteins, leading to ER stress (Zhang et al., 2015; Xu et al., 2005; Barrett et al., 2007). This event triggers the unfolded protein response (UPR) resulting in the activation of pro-apoptotic cascades (Sano and Reed, 2013; Madeo and Kroemer, 2009). The three transmembrane receptor pathways activated during ER stress are (1) inositol-requiring protein 1 alpha (IRE1α, also known as ERN1), (2) RNA-dependent protein kinase-like ER kinase (PERK) and (3) activating transcription factor 6 (ATF6) (Sano and Reed, 2013). The primary function of these receptors is to alleviate stress and restore normal cell function by reducing misfolded proteins. However, under prolonged ER stress, these pathways become over-activated, resulting in incorrect signaling and thus leading to apoptosis (Tabas and Ron, 2011). Among the three stress sensor receptors, IRE1α is the most conserved receptor (Ron and Walter, 2007).

IRE1α has a kinase and endoribonuclease domain that, upon activation, initiates the splicing of the transcription factor X-box-binding protein 1 (XBP1), making it a potent inducer of pro-apoptotic signaling (Hetz and Glimcher, 2009). In this study, we focused on identifying a novel role for Bax inhibitor-1 (BI-1) in the control of the IRE1α apoptotic signaling pathway after HI injury.

The BI-1 protein, also referred to as transmembrane BAX inhibitor motif­-containing 6 (TMBIM6), is a cell death regulator whose sequence is highly conserved among species (Chae et al., 2003; Hückelhoven, 2004; Reimers et al., 2008). BI-1, encoded by the *TMBIM6* gene (Xu and Reed, 1998; Li et al., 2014; Rojas-Rivera and Hetz, 2015), primarily resides within the ER membrane and is part of the TMBIM family involved in cytoprotection (Watanabe and Lam, 2009; Xu and Reed, 1998; Iwata et al., 2011). Studies have shown its involvement in suppressing intrinsic cell death (Xu and Reed, 1998; Xu et al., 2008), ER stress (Chae et al., 2004; Lisbona et al., 2009), ischemia (Bailly-Maitre et al., 2006; Dohm et al., 2006; Krajewska et al., 2011) and early brain injury after subarachnoid hemorrhage (Liu et al., 2018; Shi et al., 2018).

The anti-apoptotic signaling pathway of BI-1 is not entirely understood, but it may involve regulation of (1) the ER intraluminal Ca^2+^ concentration and its release, and (2) the UPR, via inhibition of IRE1α. At the mechanistic level, BI-1 has been shown to inhibit IRE1α via a direct interaction (Lisbona et al., 2009; Bailly-Maitre et al., 2010). Therefore, in this study we examined more closely the inhibitory effects of BI-1 in the UPR response in an *in vivo* neonatal HI rat model, explicitly focusing on the IRE1α branch. Furthermore, we employed an *in vitro*, oxygen-glucose deprivation (OGD) model, to establish the cell specificity of our *in vivo* findings as well as to investigate other potential signaling pathways that may be involved in the protective properties of BI-1.

Our specific objective was to determine whether overexpression of the BI-1 protein, via administration of a human adenoviral-TMBIM6 (Ad-TMBIM6) vector, would attenuate the morphological and neurological consequences of post-neonatal HI through attenuation of ER-stress-induced pathways.

## RESULTS

### Temporal changes in the expression levels of endogenous BI-1, IRE1α, XBP1 and CHOP post-HI

**Fig. 1.**
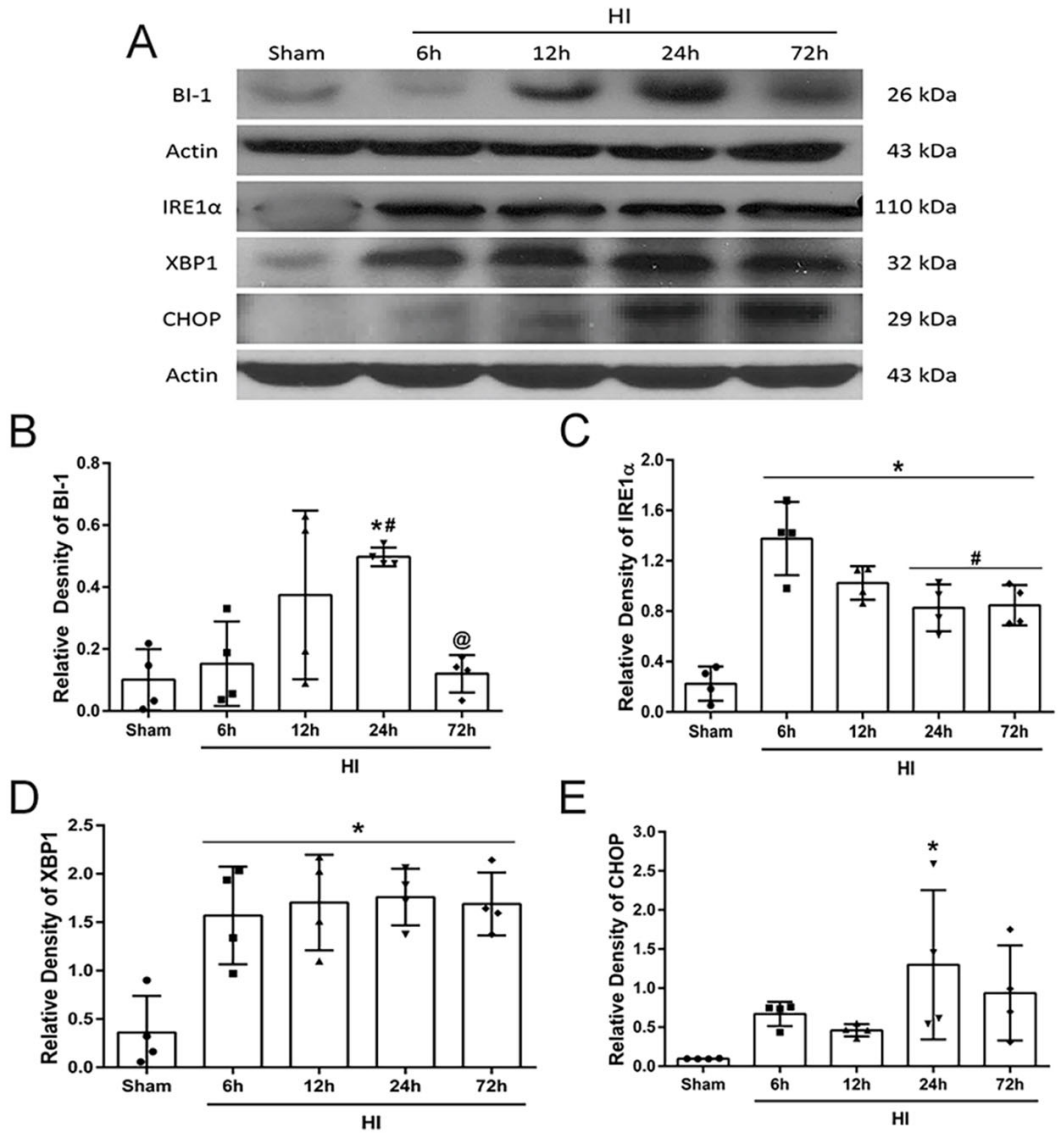
**Expression levels of endogenous BI-1, IRE1α, XBP1 and CHOP post-HI.** (A) Representative immunoblots showing protein expression levels in ipsilateral hemispheric tissue from 10-day-old neonatal rats. (B–E) Quantitative analysis of BI-1 (B), IRE1α (C), XBP1 (D) and CHOP (E) time-dependent expression after HI (band density relative to actin). Data presented as mean±s.d.; *P<0.05 versus sham; ^#^*P*<0.05 versus 6 h, ^@^*P*<0.05 versus 24 h; *n*=4. One-way ANOVA followed by Tukey multiple-comparison post hoc analysis.

In ipsilateral hemispheric brain tissue samples from 10-day-old neonatal rats subjected to HI, BI-1 expression levels increased over time, peaking at 24 h and then returning to sham levels by 72 h post-HI (Fig. 1A,B). IRE1α and XBP1 expression levels significantly increased at 6 h post-HI and remained elevated until 72 h post-HI (Fig. 1A,C,D). CHOP levels were significantly increased at 24 h post-HI compared to sham (Fig. 1A,E). Please refer to Table S1 for detailed statistical analysis.

### Ad-TMBIM6 administered at 48 h pre-HI reduced infarct area at 72 h post-HI

**Fig. 2.**
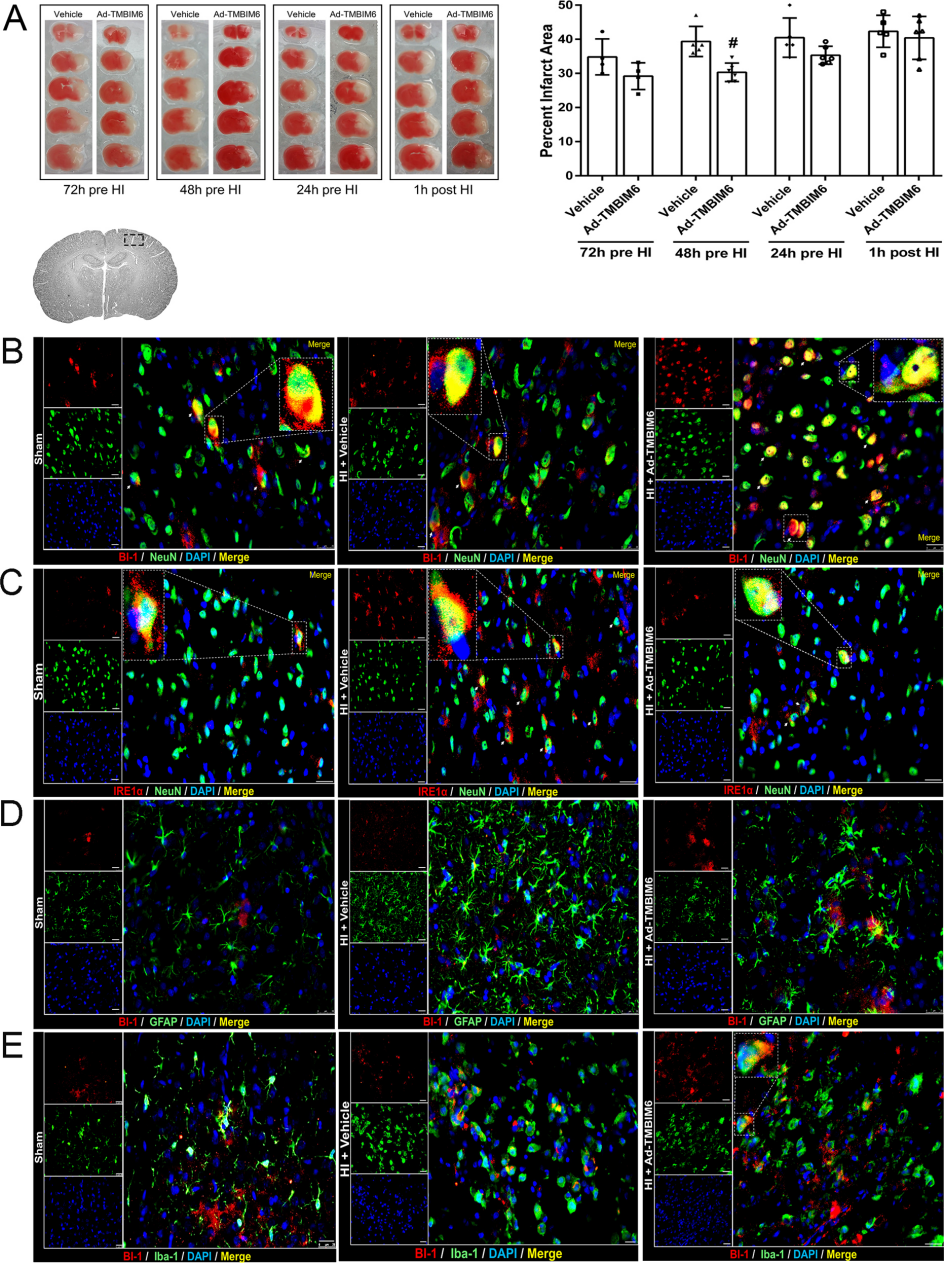
**Ad-TMBIM6 administered at 48 h pre-HI reduced percentage infarcted area and showed localization to neurons and microglia at 72 h post-HI.** (A) Representative images of TTC-stained sections and quantification of percentage infarcted area at 72 h post-HI in brain tissue from neonatal rats expressing BI-1 through adenoviral transduction with Ad-TMBIM6. Data presented as mean±s.d.; *P<0.05 versus sham; ^#^*P*<0.05 versus vehicle, *n*=6. One-way ANOVA followed by Tukey multiple-comparison post hoc analysis. (B–E) Representative microphotographs of double immunofluorescence staining of BI-1 (red) with neurons (NeuN, green) (B), IRE1α (red) with neurons (NeuN, green) (C), BI-1 (red) with astrocytes (GFAP, green) (D) and BI-1 (red) with microglia (Iba-1, green) (E) in the peri-infarcted area at 72 h post-HI. Blue, DAPI. *n*=1/group. Merged images show localization of BI-1 or IRE1α to neurons, astrocytes or microglia. There was minimal localization of BI-1 to astrocytes. Scale bars: 50 μm.

To identify the best time for administration of the Ad-TMBIM6 vector in our *in vivo* model, we tested four time points; 72 h, 48 h and 24 h pre-HI and 1 h post-HI. Ad-TMBIM6 administered 48 h before HI significantly reduced the infarct area compared to the vehicle-treated group (Fig. 2A). Best dose of viral vector was determined from preliminary experiments (data not shown). Please refer to Table S2 and Table S9 for detailed statistical analysis.

### Histological staining at 72 h post-HI

Immunofluorescence staining showed a higher expression of BI-1 on neurons in the Ad-TMBIM6 treatment group when compared to vehicle (Fig. 2B). Furthermore, IRE1α was decreased in the Ad-TMBIM6 treatment group when compared to vehicle; IRE1α also localized to neurons in the sham, vehicle and treated groups (Fig. 2C). In addition, we observed minimal localization of BI-1 to astrocytes in all groups (Fig. 2D). BI-1 showed localization to microglia (Fig. 2E). Cell types (neuron, astrocyte and microglia) were stained green. BI-1 and IRE1α were stained red. DAPI is shown in blue. The merged images show the localization of BI-1 or IRE1α to neurons, astrocytes and microglia. To confirm localization of BI-1 to microglia, we have also stained samples investigated for BI-1 with a second microglial marker, CD11b/c (Fig. S1).

### Ad-TMBIM6 improved long-term neurological function at 4 weeks post-HI

**Fig. 3.**
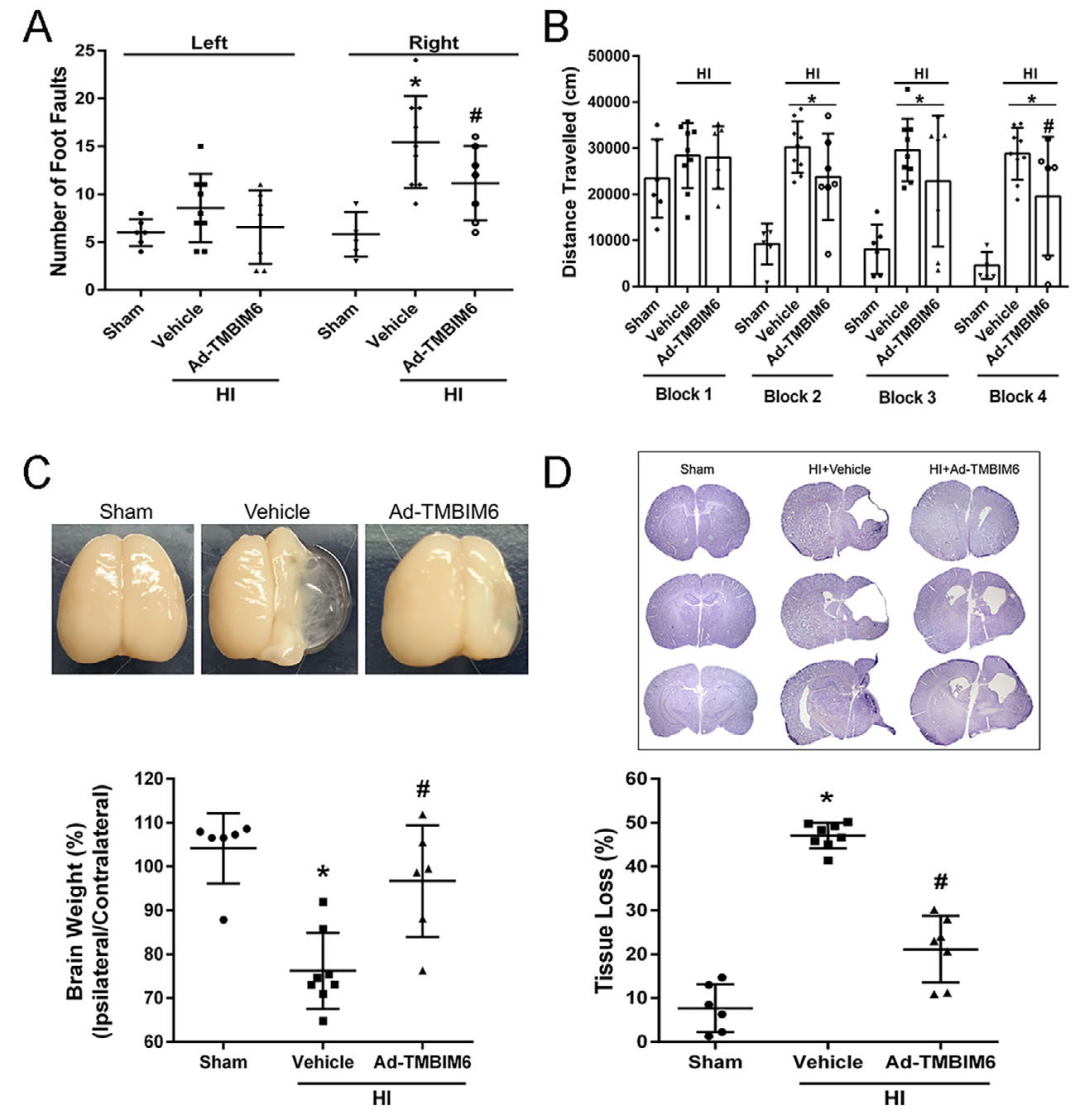
**Expression of BI-1 improved long-term neurological deficits at 4 weeks post-HI.** (A,B) In rat pups expressing BI-1 through treatment with Ad-TMBIM6 , long-term motor, learning and memory were assessed via foot fault test (A) and water maze test (B) at 4 weeks post-HI. (C,D) Quantitative analysis of percentage of weight of ipsilateral and contralateral regions out of total brain weights (C) and percentage of tissue loss (D) at 4 weeks post-HI. Data presented as mean±s.d.; *P<0.05 versus sham; ^#^*P*<0.05 versus vehicle; *n*=6–9. One-way ANOVA followed by Tukey multiple-comparison or Holm–Sidak post hoc analysis.

To test the effects of Ad-TMBIM6 treatment at 48 h pre-HI on the long-term neurological impairments induced by neonatal HI, neurological function was assessed by means of foot fault and water maze tests at 4 weeks post-HI. In motor and memory tests, the vehicle group performed significantly worse compared to sham animals. The Ad-TMBIM6-treated group had significantly improved sensorimotor coordination as displayed by the fewer number of foot faults (Fig. 3A). In the water maze test, compared to sham group, the vehicle group demonstrated substantial memory impairment and learning abilities in terms of the distance traveled to find the platform (Fig. 3B). However, Ad-TMBIM6 significantly improved both memory and learning function compared to vehicle (Fig. 3B). Furthermore, administration of Ad-TMBIM6 significantly reduced brain atrophy versus vehicle (Fig. 3C). Nissl staining showed that Ad-TMBIM6 attenuated the percentage of brain tissue loss (Fig. 3D). Please refer to Table S3 and Table S10 for detailed statistical analysis.

### BI-1 siRNA and IRE1α CRISPR activation plasmid reversed Ad-TMBIM6 protective effects at 72 h post-HI

**Fig. 4.**
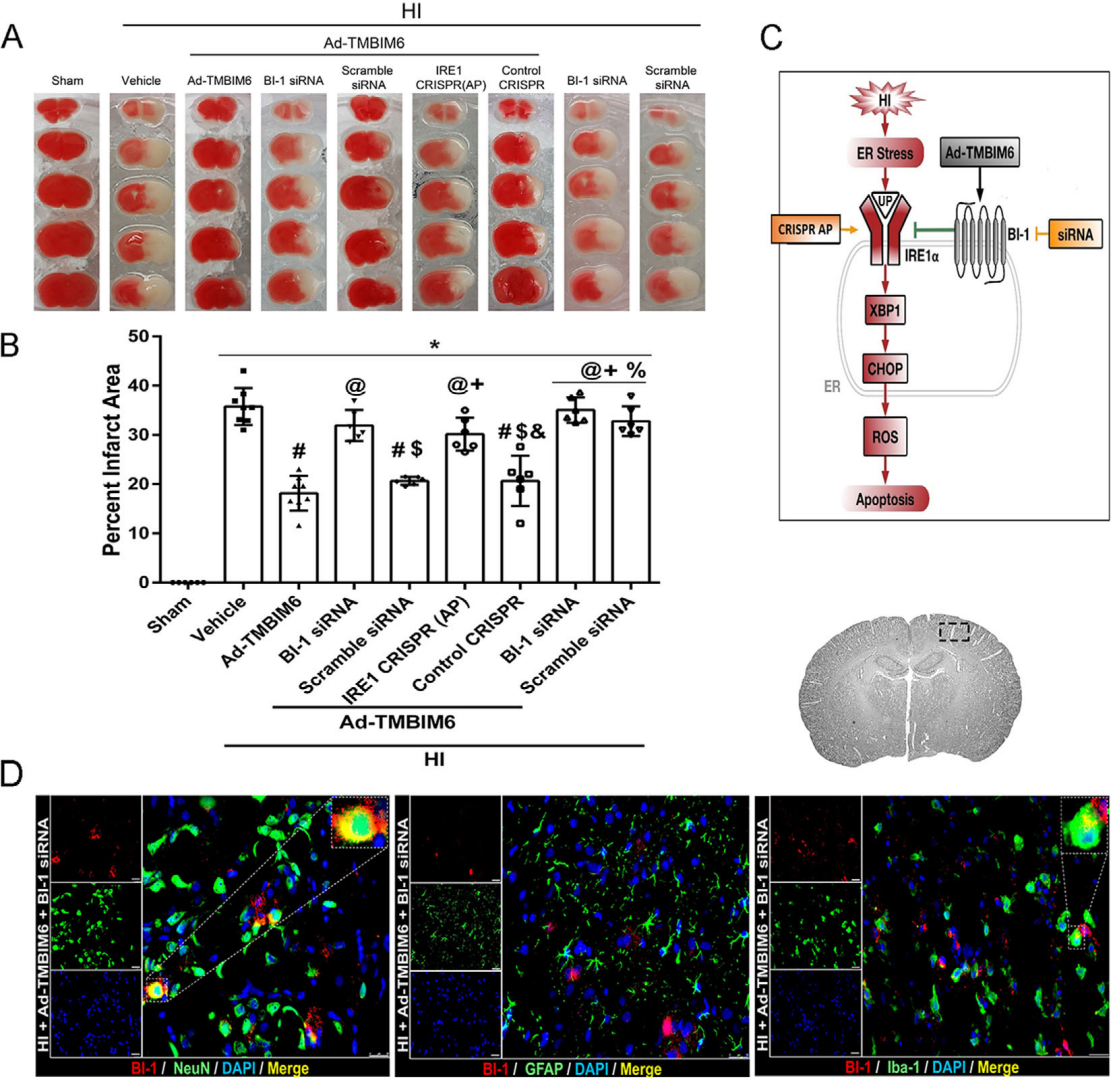
**Effects of BI-1 siRNA and IRE1α CRISPR activation plasmid on infarct area at 72 h post-HI.** (A,B) Representative images of infarcted tissue (A) and quantification of percentage infarcted area (out of total brain area) (B) at 72 h post-HI. Data presented as mean±s.d.; *P<0.05 versus sham; ^#^*P*<0.05 versus vehicle; ^@^*P*<0.05 versus Ad-TMBIM6; ^$^*P*<0.05 versus BI-1 siRNA; ^+^*P*<0.05 versus Ad-TMBIM6+scramble siRNA; ^&^*P*<0.05 versus IRE1α CRISPR AP; ^%^*P*<0.05 versus control CRISPR. *n*=6/group. One-way ANOVA followed by Tukey multiple-comparison post hoc analysis. (C) Representative schematic of BI-1/IRE1α/XBP1/CHOP signaling pathway using the intervention groups (BI-1 siRNA and IRE1α CRISPR activation plasmid) to study the mechanism. (D) Representative immunofluorescence staining of BI-1 (red) with neurons (NeuN, green), astrocytes (GFAP, green) and microglia (Iba-1, green) after siRNA administration. Representative picture demonstrating brain region from where immunofluorescence pictures were obtained is shown upper right. *n*=1/group. Scale bars: 50 μm.

To evaluate whether pathway interventions can affect apoptosis and increase the size of the infarcted area, we silenced BI-1 (using siRNA against BI-1) and upregulated IRE1α expression [using an IRE1α CRISPR activation plasmid (AP)]. The results showed that, for animals treated with Ad-TMBIM6, both BI-1 siRNA and IRE1α CRISPR AP significantly increased the relative size of infarcted area (as a percentage of total brain area) compared to the Ad-TMBIM6-treated group, thus reversing the protective effects of BI-1 (Fig. 4A,B). Control groups, treated with scramble siRNA and control CRISPR AP, did not exhibit exacerbated damage when compared to the Ad-TMBIM6-treated group. Furthermore, treatment with BI-1 siRNA only (without Ad-TMBIM6) further exacerbated the injuries when compared to the Ad-TMBIM6 treatment group. Please refer to Table S4 for detailed statistical analysis. Immunofluorescence staining showed that BI-1 siRNA reduced expression of BI-1 in neurons and microglia (Fig. 4D).

### BI-1 exerted its neuroprotective effects via the IRE1α–XBP1–CHOP pathway at 72 h post-HI

**Fig. 5.**
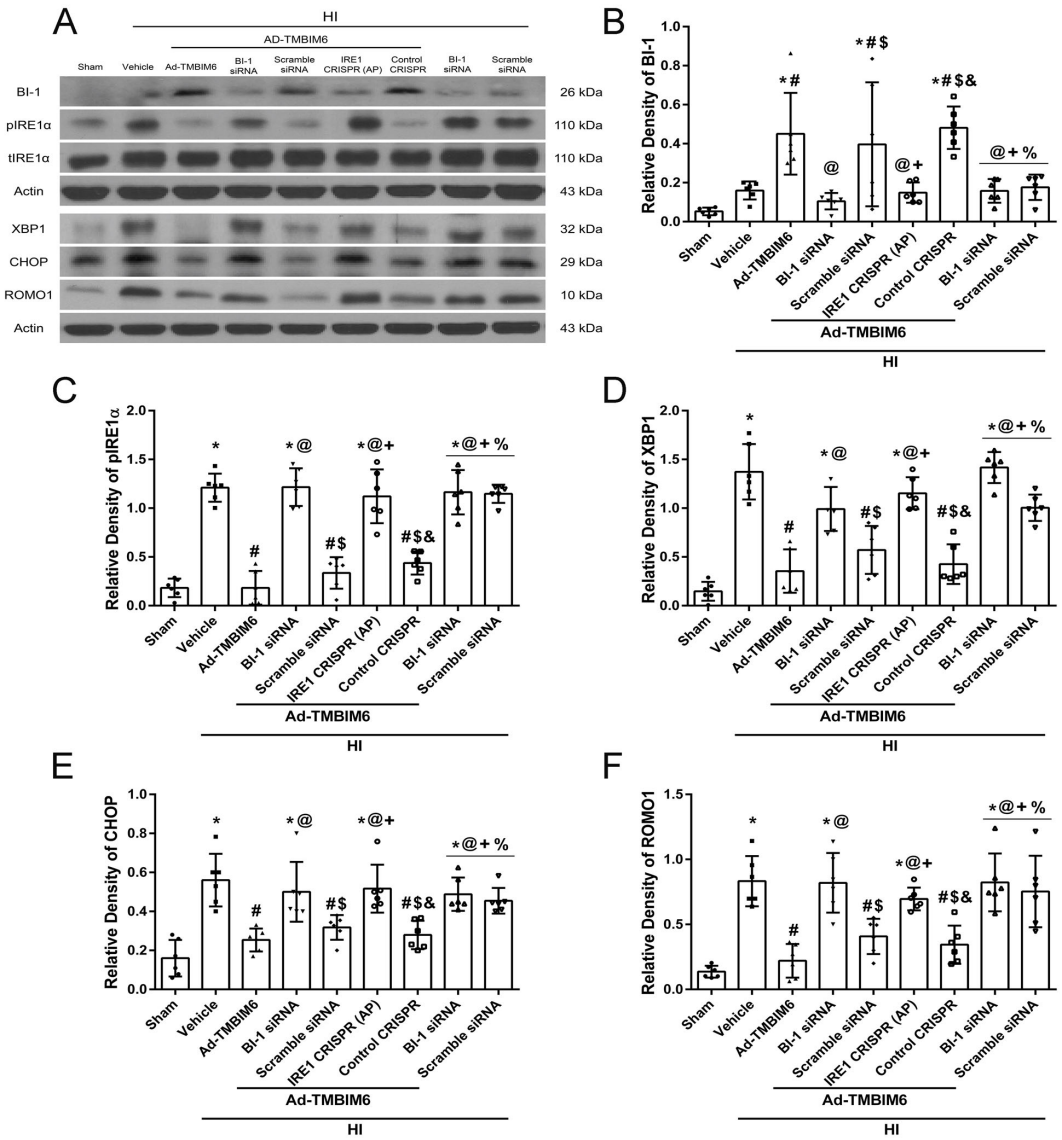
**BI-1 exerted its protective effects via the IRE1α–XBP1–CHOP pathway at 72 h post-HI.** (A) Representative immunoblots of protein expression levels in ipsilateral hemispheric tissue from 10-day-old rats at 72** **h post-HI. (B–F) Quantitative analysis of BI-1 (B), pIRE1α (C), XBP1 (D), CHOP (E) and ROMO1 (F) expression levels (band density relative to actin). Data presented as mean±s.d.; *P<0.05 versus sham; ^#^*P*<0.05 versus vehicle; ^@^*P*<0.05 versus Ad-TMBIM6; ^$^*P*<0.05 versus BI-1 siRNA; ^+^*P*<0.05 versus Ad-TMBIM6+scramble siRNA; ^&^*P*<0.05 versus IRE1α CRISPR AP; ^%^*P*<0.05 versus control CRISPR; ^^^*P*<0.05 versus Ad-TMBIM6+BI-1 siRNA; ^ε^*P*<0.05 versus BI-1 siRNA-only group. *n*=6/group. One-way ANOVA followed by Tukey multiple-comparison post hoc analysis.

To test the underlying mechanism by which BI-1 attenuates apoptosis, animals were subjected to the following treatment groups: sham, vehicle, Ad-TMBIM6, Ad-TMBIM6+BI-1 siRNA, Ad-TMBIM6+scramble siRNA, Ad-TMBIM6+IRE1α CRISPR AP, AD-TMBIM6+control CRISPR, BI-1 siRNA-only and scramble siRNA-only groups. The expression levels of BI-1 were significantly increased in ipsilateral hemispheric tissue from the Ad-TMBIM6 treatment group, Ad-TMBIM6+scramble siRNA and in the Ad-TMBIM6+control CRISPR groups, while the BI-1 siRNA and IRE1α CRISPR AP groups showed the opposite effects (Fig. 5A,B). BI-1 siRNA-only and scramble siRNA-only groups had similar BI-1 expression levels as that of the vehicle group. Ad-TMBIM6 treatment significantly reduced the expression of phosphorylated IRE1α (pIRE1α), XBP1, CHOP and ROMO1 [a modulator that induces production of reactive oxygen species (ROS)] while BI-1 siRNA and IRE1α CRISPR AP reversed the protective effects of BI-1 and also elevated expression of pro-apoptotic pathway proteins (Fig. 5A,C–F). No significant difference was observed in total IRE1α expression levels (data not shown). Please refer to Table S5 for detailed statistical analysis. In addition, we have provided immunofluorescence images to show the extent of the spread of ER stress marker, IRE1α, within microglia around the peri-infarcted area from three different tissue samples (Fig. S2).

### Overexpression of BI-1 suppressed pro-apoptotic proteins and attenuated neuronal apoptosis at 72 h post-HI

**Fig. 6.**
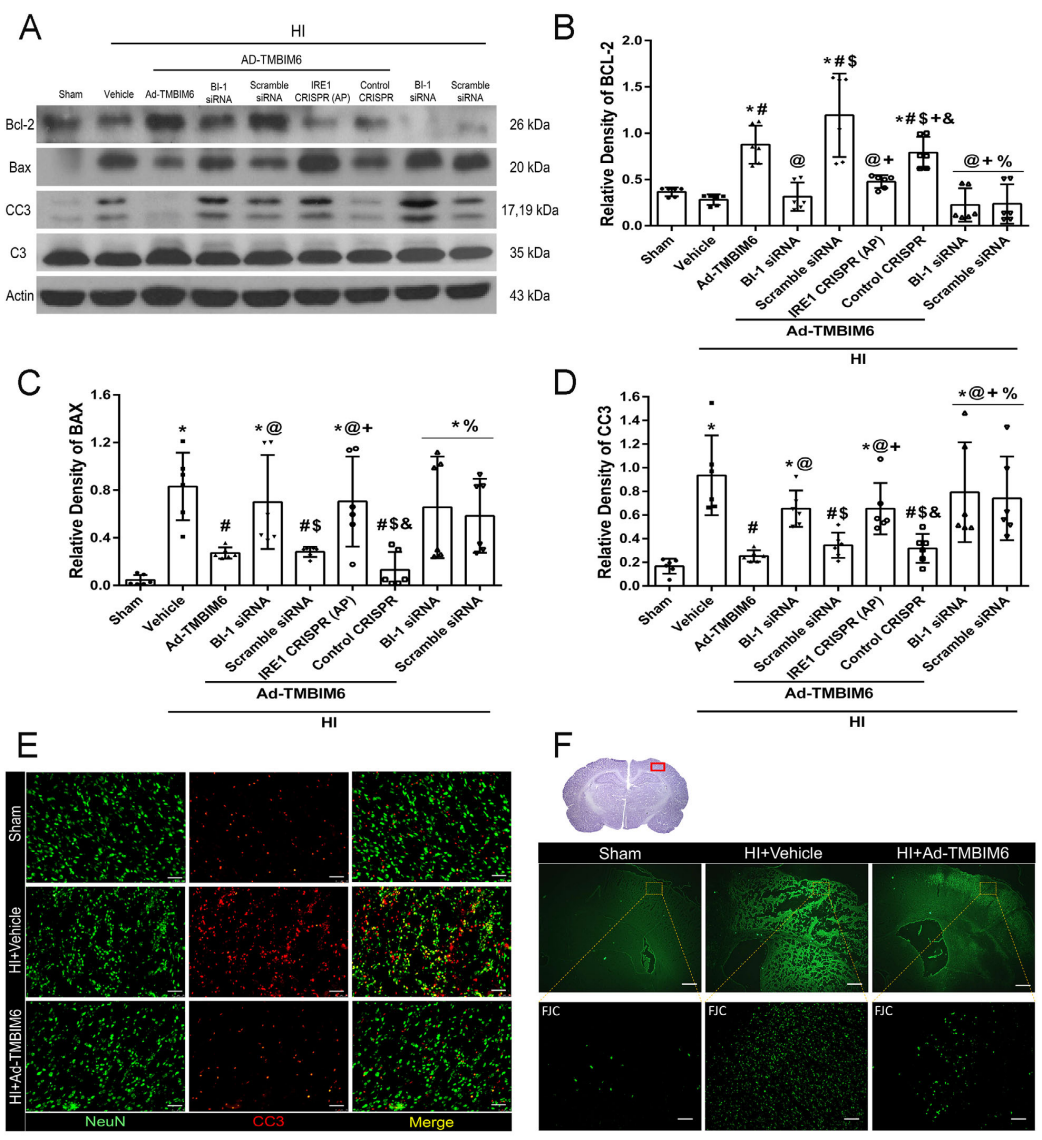
**Overexpression of BI-1 attenuated apoptosis at 72 h post-HI.** (A) Representative immunoblots of protein expression levels in ipsilateral hemispheric tissue from 10-day-old rats at 72** **h post-HI. (B–D) Quantification of Bcl-2 (B), BAX (C) and CC3 (D) expression level data (band density relative to actin). Data presented as mean±s.d.; *P<0.05 versus sham; ^#^*P*<0.05 versus vehicle; ^@^*P*<0.05 versus Ad-TMBIM6; ^$^*P*<0.05 versus BI-1 siRNA; ^+^*P*<0.05 versus Ad-TMBIM6+scramble siRNA; ^&^*P*<0.05 versus IRE1α CRISPR AP; ^%^*P*<0.05 versus control CRISPR. *n*=6/group. One-way ANOVA followed by Tukey multiple-comparison, Holm–Sidak or Dunnett's post hoc analysis. (E) Representative microphotographs of positively CC3-stained (red) neurons (NeuN, green) from 10-day-old rats at 72** **h post-HI. (F) Representative microphotographs of Fluoro-Jade C (FJC)-positive degenerating neurons in the peri-infarcted area. *n*=1/group. Scale bars: 100 µm.

To evaluate the effects of BI-1 overexpression on apoptosis by Ad-TMBIM6, western blotting was performed to quantify expression levels BCL-2, BAX, cleaved caspase 3 (CC3) and caspase 3 in ipsilateral hemispheric tissue from vehicle- and Ad-TMBIM6-treated animals. The data showed that Ad-TMBIM6 treatment significantly upregulates anti-apoptotic marker BCL-2 (Fig. 6A,B) while attenuating the expression of pro-apoptotic markers BAX and CC3 (Fig. 6A,C,D). In addition, administration of BI-1 siRNA or IRE1α CRISPR AP reversed the protective effects of Ad-TMBIM6, leading to significantly increased levels of pro-apoptotic markers (BAX and CC3) and downregulating the anti-apoptotic marker BCL-2 (Fig. 6A–D). There was no significant difference in total caspase 3 expression levels (data not shown). Please refer to Table S6 for detailed statistical analysis.

Since HI results in neuronal degeneration and apoptosis, we labeled CC3 with Fluoro-Jade C, and evaluated staining levels to measure the neuroprotective effects of Ad-TMBIM6. Immunofluorescence staining showed higher expression of CC3, which localized in neurons, in the vehicle group compared to sham or the Ad-TMBIM6 groups (Fig. 6E). Fluoro-Jade C staining showed a higher number of neurons undergoing apoptosis in vehicle group compared to sham or Ad-TMBIM6 treatment groups (Fig. 6F).

### Optimizing time for OGD and Ad-TMBIM6 vector multiplicity of infection for successful transfection of PC12 cells as an *in vitro* OGD model

**Fig. 7.**
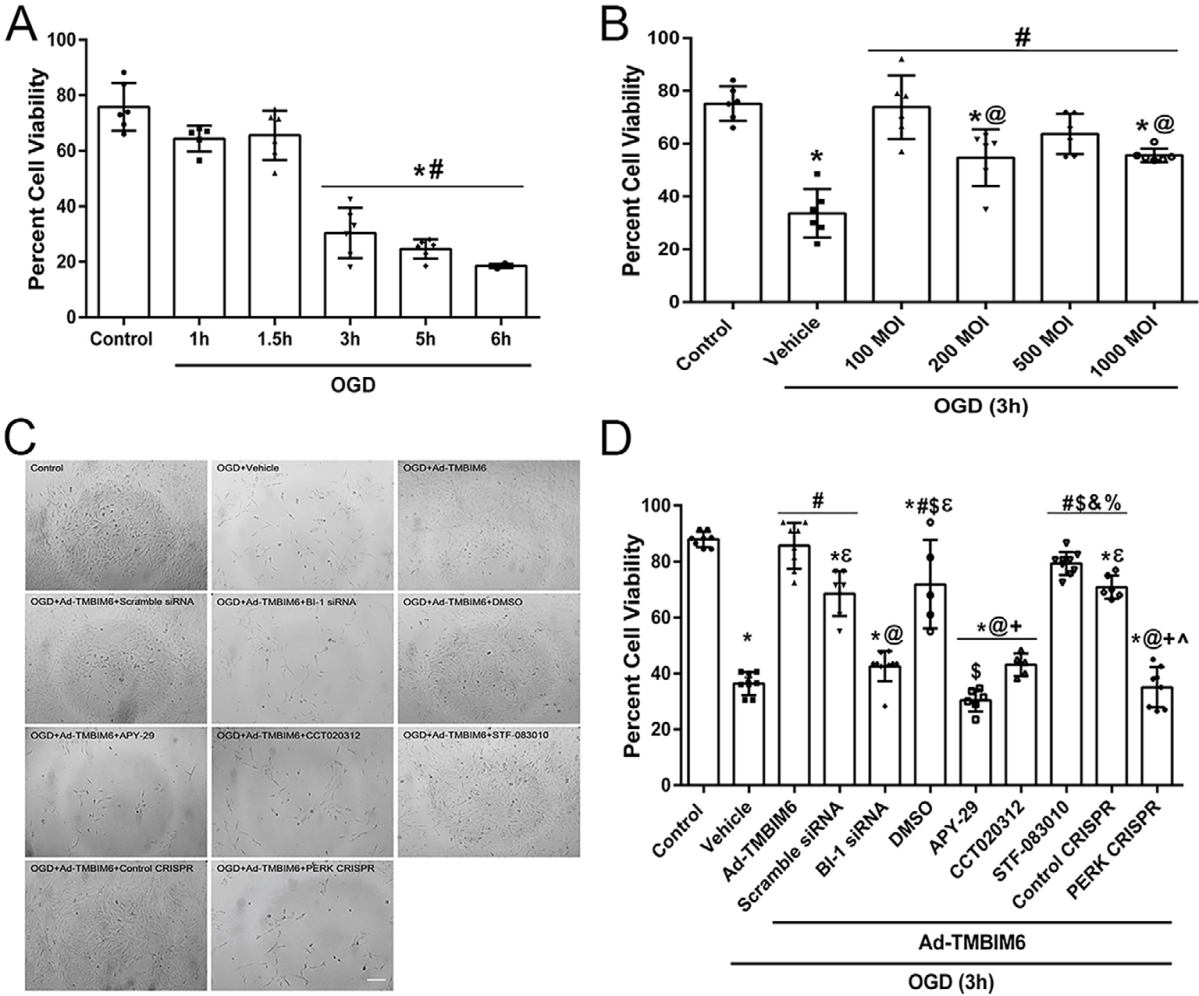
**Percentage cell viability significantly improved after transfection of PC-12 cells with Ad-TMBIM6 at MOI=100 in an *in vitro* OGD model.** (A) Quantification of data shows time-dependent decrease in percentage cell viability for cultured PC12 cells when exposed to 1 h, 1.5, 3 h, 5 h and 6 h in OGD. (B) Quantification analysis of percentage cell viability of PC12 cells when exposed to varying concentrations (MOI) of viral vector. (C) Representative images of cell morphology and density of cells after OGD in all groups. Scale bar: 100 µm. (D) Quantification analysis of percentage cell viability with pathway interventions. Data presented as mean± s.d.; *P<0.05 versus control; ^#^*P*<0.05 versus vehicle; ^@^*P*<0.05 versus Ad-TMBIM6+scramble siRNA (D) or versus MOI=100 (B); ^ε^*P*<0.05 versus Ad-TMBIM6 only; ^$^*P*<0.05 versus BI-1 siRNA; ^&^*P*<0.05 versus APY-29; ^+^*P*<0.05 versus DMSO; ^%^*P*<0.05 versus CCT020312; ^^^*P*<0.05 versus STF-083010 and control CRISPR. *n*=5–6/group. One-way ANOVA followed by Tukey multiple-comparison post hoc analysis.

Data showed a time-dependent decrease in percentage cell viability in cultured rat PC12 cells when exposed to variable periods of OGD. Cells were exposed to five different OGD periods ranging from 1 h to 6 h. Percentage cell viability was significantly reduced when compared to untreated control at 3 h, 5 h and 6 h (Fig. 7A). The optimal time for OGD exposure was chosen to be 3 h to best represent the severity of injury in our *in vivo* HI model.

Optimal multiplicity of infection (MOI) of the Ad-TMBIM6 vector was shown to be MOI=100, as cells infected at this level showed significantly improved cell viability compared to vehicle-treated cells and cells exposed to MOI=200 or MOI=1000 after 3 h of OGD (Fig. 7B). Please refer to Table S7 for detailed statistical analysis.

### Determining the effect of Ad-TMBIM6 on percentage cell viability in an *in vitro* OGD model

To test the role of the IRE1α signaling pathway in the protective mechanism of BI-1, PC12 cells were transfected with BI-1 siRNA, and treated with an IRE1α agonist (APY-29) and an IRE1α antagonist (STF-083010) in combination with Ad-TMBIM6. To test the involvement of the PERK signaling pathway in the protective mechanism of BI-1, PC12 cells were subjected to PERK CRISPR knockdown and treated with CCT020312 (PERK agonist) together with Ad-TMBIM6. Cell viability data indicated that vehicle-treated cells exposed to 3 h of OGD showed a significantly higher percentage of cell death when compared to the control and OGD+Ad-TMBIM6 groups (Fig. 7C,D). Treatment with BI-1 siRNA, APY-29 (20 µM) or CCT020312 (10 µM) reversed the protective effects of BI-1, as seen from the significantly lower percentage of cell viability (Fig. 7D). Ad-TMBIM6+PERK CRISPR did not improve cell viability when compared to the OGD-only group. By contrast, adding STF-083010 (20 µM) to the Ad-TMBIM6 group led to significantly improved cell viability (∼85% viable cells) when compared to the PERK CRISPR or OGD (both ∼40% viable cells) groups.

In the control groups, OGD+Ad-TMBIM6+scramble siRNA, OGD+Ad-TMBIM6+DMSO or OGD+Ad-TMBIM6+control CRISPR treatment led to significantly improved cell viability when compared to vehicle group or to OGD+Ad-TMBIM6 with APY-29, CCT020312 or PERK CRISPR, but did not restore cell viability up to control or OGD+Ad-TMBIM6-only group levels. Please refer to Table S7 for detailed statistical analysis.

### Establishing the anti-apoptotic effects of BI-1 via the IRE1α signaling pathway in an *in vitro* OGD model

**Fig. 8.**
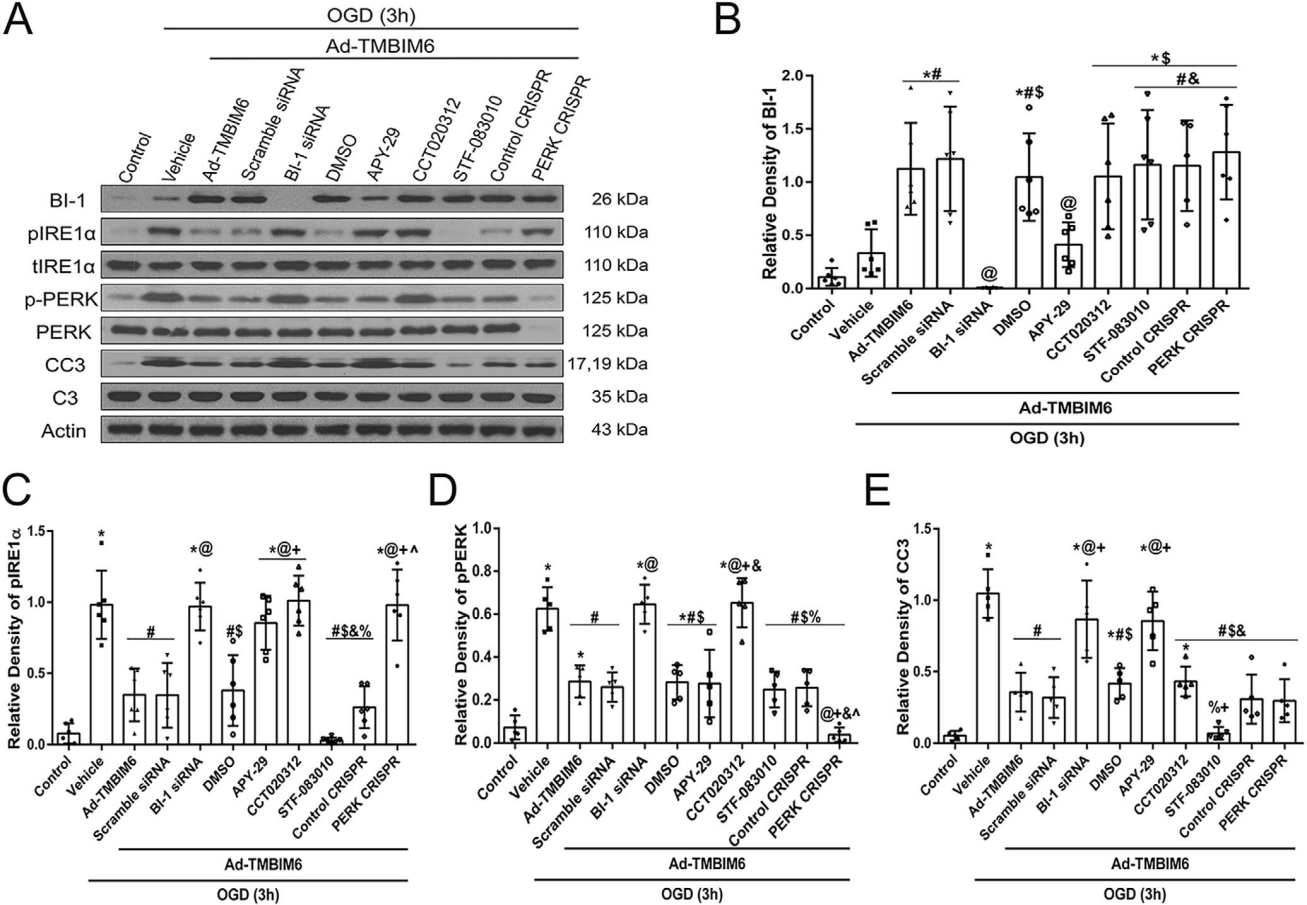
**BI-1 exerted its anti-apoptotic effects via inhibition of IRE1α in an *in vitro* OGD model.** (A) Representative immunoblots of protein expression levels in PC12 cultured cells. Quantification of BI-1 (B), pIRE1α (C), pPERK (D) and CC3 (E) expression levels (band density relative to actin). Data presented as mean±s.d.; *P<0.05 versus control; ^#^*P*<0.05 versus vehicle; ^@^*P*<0.05 versus Ad-TMBIM6 and Ad-TMBIM6+scramble siRNA; ^$^*P*<0.05 versus BI-1 siRNA; ^&^*P*<0.05 versus APY-29; ^+^*P*<0.05 DMSO; ^%^*P*<0.05 versus CCT020312; ^^^*P*<0.05 versus STF-083010 and control CRISPR. *n*=5–6/group. One-way ANOVA followed by Tukey multiple-comparison post hoc analysis.

To test the underlying mechanism by which BI-1 attenuated apoptosis, both IRE1α agonist (APY-29) and antagonist (STF-083010) were used, in addition to PERK agonist (CCT020312) and knockdown (PERK CRISPR knockdown), in PC12 cells subjected to Ad-TMBIM6 and OGD treatment. These treatments allowed the manipulation of both pathways in order to determine the predominant one. Quantification data showed that in cells treated with OGD+Ad-TMBIM6, BI-1 expression levels were significantly increased in the Ad-TMBIM6-only treatment group and in the control groups (scramble siRNA, DMSO and control CRISPR), while treatment with BI-1 siRNA and APY-29 reversed those effects (Fig. 8A,B). Cells treated with CCT020312 and PERK CRISPR, as well as STF-083010, showed similar BI-1 expression levels to that of the Ad-TMBIM6-only treatment group (Fig. 8A,B). Intervening at the PERK receptor did not seem to alter BI-1 expression levels since CCT020312 did not affect BI-1 levels, whereas intervening at IRE1α with APY-29 significantly reduced BI-1's levels (Fig. 8B). In the Ad-TMBIM6-only and Ad-TMBIM6 with scramble siRNA, DMSO or control CRISPR groups, pIRE1α, phosphorylated PERK (pPERK) and CC3 expression levels were significantly reduced (Fig. 8C–E) compared to vehicle. Meanwhile, treatment with BI-1 siRNA, APY-29, CCT020312 and PERK CRISPR increased pIRE1α expression levels (Fig. 8C). Expression levels of pPERK were significantly upregulated after OGD in vehicle, BI-1 siRNA and CCT020312 groups when compared to the rest of the groups (Fig. 8D). While CC3 expression was significantly reduced in the Ad-TMBIM6-only and control groups (Ad-TMBIM6 with scramble siRNA, DMSO and control CRISPR), activation of PERK with CCT020312 did not significantly upregulate CC3 when compared to treatment or control groups (Fig. 8E); in fact, CCT020312 significantly decreased CC3 expression levels. Furthermore, APY-29 treatment significantly upregulated CC3 compared to CCT020312. In addition, STF-083010 was more effective at reducing CC3 compared to PERK CRISPR (Fig. 8E). In addition, we have included representative western blot bands showing expression densities of BI-1, p-IRE1α, p-PERK and CC3 from our primary neuronal cell cultures (Fig. S3). No difference was observed in the expression of the above-listed proteins between primary neuronal cell cultures and PC12 cells. Please refer to Table S8 for statistical analysis.

## DISCUSSION

Despite several treatments being available for hypoxic-ischemic (HI) injury, 40–50% of treated patients still experience persistent neurodevelopmental deficits (Zanelli et al., 2011; Barrett et al., 2007), thus the need to explore alternative treatment strategies. A crucial step towards the development of novel therapeutic modalities is first to understand the cellular and molecular mechanisms involved and how to manipulate them (Volpe, 2001; Bracewell and Marlow, 2002; van Handel et al., 2007).

ER stress is a major pathology encountered post-HI injury, which is responsible for the control of correct protein folding and function (Zhang et al., 2015). A variety of disturbances and stresses, such as ischemic events, can modify ER function and result in the accumulation of unfolded proteins, thus triggering the UPR (Rissanen et al., 2006; Xu et al., 2005). Under prolonged stress, such as after HI injury, accumulation of unfolded proteins leads to the activation of stress sensor receptors that trigger pro-apoptotic signaling, leading to cell death (Oyadomari and Mori, 2004; Madeo and Kroemer, 2009; Chaudhari et al., 2014; Zhang et al., 2015; Carloni et al., 2014; Xu et al., 2005). The three main receptors activated are IRE1α, PERK and ATF6 (Sano and Reed, 2013). Among these three UPR proteins, IRE1α is one of the key molecules, as it is responsible for cell fate by directly binding to misfolded proteins and triggering a signaling response (Sano and Reed, 2013; Gardner and Walter, 2011). Accumulation of unfolded proteins stimulates autophosphorylation of the IRE1α kinase domain, which activates the RNase to splice XBP1. This acts as a potent transcription factor that triggers pro-apoptotic signaling mechanisms (Hetz et al., 2011). Concomitant with previous studies, our results showed that after HI injury, endogenous expression levels of pIRE1α and its downstream pro-apoptotic molecules, XBP1 and CHOP, increased in a time-dependent manner (Fig. 1).

Therefore, in this study, we focused on ER-stress-induced signaling pathways. Specifically, our primary goal was to identify a novel role for BI-1 and ER stress in the pathophysiology of neonatal HI by targeting ER stress sensor receptors in an *in vivo* HI rat model and in an *in vitro* oxygen-glucose deprivation (OGD) model.

BI-1 has been characterized in various *in vitro* injury mechanisms as a pro-survival protein (Robinson et al., 2011; Lisbona et al., 2009; Lee et al., 2007; Chae et al., 2004; Bailly-Maitre et al., 2010, 2006; Castillo et al., 2011). BI-1 is a small hydrophobic protein that resides on the ER membrane that was first discovered in yeast (Xu and Reed, 1998). Initial studies showed that BI-1 expression levels varied during various stages of life, such as high upregulation during times of high apoptosis (Chae et al., 2004; Jean et al., 1999), thus suggesting that there may be a close link between BI-1 and cell death. *In vitro* studies in cultured cells began to examine the anti-apoptotic effects of BI-1, which were later translated to an *in vivo* mouse model (Chae et al., 2004; Bailly-Maitre et al., 2006; Krajewska et al., 2011; Ma et al., 2014). Since its initial discovery, more and more emerging studies have provided evidence that the protective effects of BI-1 are most prominent during ER stress. Several *in vitro* studies have shown that BI-1 protects against the effects of thapsigargin, tunimycin (Chae et al., 2004) and OGD. Furthermore, cells overexpressing BI-1, after exposure to ER stress inducers, did not show characteristics of apoptotic changes (Chae et al., 2004), whereas cells that lacked BI-1 exhibited sensitivity to ER stress inducers. From *in vitro* cultured cell experiments, studies moved onto using transgenic animals overexpressing BI-1. These studies demonstrated that cells cultured from these transgenic animals were protected from various stresses (Chae et al., 2004). By contrast, cells cultured from BI-1-deficient animals exhibited much more sensitivity to ER stress (Krajewska et al., 2011).

Thus far, despite the numerous *in vitro* studies into the anti-apoptotic roles of BI-1, there is limited *in vivo* data to show the protective effect and signaling mechanisms of BI-1 in stroke-related models. Recently, limited studies of *in vivo* models of ischemia-reperfusion, traumatic brain injury (Krajewska et al., 2011) and subarachnoid hemorrhage (Liu et al., 2018; Shi et al., 2018), either using transgenic mice that overexpress BI-1 or a lentiviral vector to introduce the gene, have shown BI-1 to confer protection by interfering with apoptotic signals and blood–brain barrier (BBB) disruption (Krajewska et al., 2011; Chae et al., 2004; Shi et al., 2018; Liu et al., 2018). However, despite the aforementioned *in vivo* studies, the mechanisms by which BI-1 confers protection remain unclear. This study will help shed light on the anti-apoptotic roles of BI-1 and its possible signaling mechanisms in an *in vivo* neonatal HI injury model. Exploring the roles of BI-1 in animals is much-needed research, owing to the dearth of *in vivo* data to support understanding of the protective properties and signaling mechanisms of BI-1.

Due to limited knowledge on BI-1, and also a lack of specific drugs targeting it, we used an adenoviral vector to overexpress the *TMBIM6* gene, giving us a robust upregulation of the BI-1 protein. However, adenoviral vectors may take 24–72 h to begin protein expression; therefore, we administered the Ad-TMBIM6 vector at multiple time points and euthanized animals at 72 h post-HI to determine the optimal time needed for the virus to replicate and begin overexpressing BI-1 (Bailly-Maitre et al., 2010). Our results indicated that administration of the Ad-TMBIM6 vector at 48 h pre-HI significantly reduced the size of the infarcted area at 72 h post-HI (Fig. 2A). However, administration of Ad-TMBIM6 before injury is not clinically translatable, thus further exploration and development of more adequate ways to target BI-1 overexpression *in vivo* are necessary.

One potential clinically translatable drug that could be used post-HI to upregulate BI-1 expression is emodin. Emodin and its active compounds, such as emodin-8-O-β-d-glucoside, are lipophilic and so can easily cross the BBB, and have also been shown to be protective (Wang et al., 2007). Emodin is a resin extract from rhubarb that has been shown to have anti-mutagenic, anti-cancer, anti-diuretic, anti-inflammatory and anti-apoptotic properties that can be administered post-injury, making it clinically translatable (Xu et al., 2005; Sano and Reed, 2013). The promise of emodin as an effective drug for HI surfaced from a study done where exogenous administration of emodin was shown to have an anti-apoptotic and anti-inflammatory effect in an acute myocardial infarction model (Wu et al., 2007). Additionally, several *in vivo* and *in vitro* studies of stroke models of cerebral ischemia (MCAO) have shown the neuroprotective properties of emodin (Guo et al., 2013; Lu et al., 2005). Furthermore, emodin has also been linked with its ability to decrease the expression of IRE1α and downstream molecules in a severe acute pancreatitis model (Wu et al., 2013). Moreover, emodin can effectively downregulate BFAR expression levels (Wang et al., 2005), hence increasing the level to which BI-1 leads to attenuation of ER stress (Krajewska et al., 2011; Lisbona et al., 2009; Bailly-Maitre et al., 2010, 2006). BFAR is an endoplasmic reticulum-associated E3 ubiquitin ligase that directly interacts with the anti-apoptotic BI-1 protein and promotes its degradation (Rong et al., 2011). Based on previous studies, a potential indirect link may exist between emodin and BI-1; however, mechanisms are unclear. Before emodin can be considered as a clinically translatable drug for the upregulation of BI-1 and its signaling pathway after HI, further research is necessary to establish the link between emodin and BI-1, and also between emodin and other potential targets of BI-1 that can be used in clinics.

In the scope of this study, we generated a robust overexpression model of BI-1 protein to help us understand the signaling mechanisms of BI-1 pertaining to inhibition of ER-stress-induced injury after hypoxic ischemic encephalopathy (HIE). Understanding these mechanisms is crucial as it will aid us in establishing how the two components interrelate and how to target them for the development of therapeutics.

Mechanistically, BI-1 has previously been described to act as a negative regulator of ER stress sensors, such as IRE1α (Lisbona et al., 2009) and to a lesser extent of PERK (Lee et al., 2007). In particular, there are several lines of evidence from *in vitro* co-immunoprecipitation studies showing a direct physical interaction between BI-1 and IRE1α (Lisbona et al., 2009). In *in vitro* models of ER stress (Sano and Reed, 2013), it was reported that BI-1 inhibited IRE1α activity by binding to IRE1α through its C-terminus and inhibiting its RNase domain (Lisbona et al., 2009), as well as inhibiting its downstream factor XBP1 (Lee et al., 2007). These observations were later confirmed by Lisbona et al. (2009), where animals deficient in BI-1 showed a significant increase in IRE1α activity and XBP1 expression. In the same study, knockdown of either IRE1α or XBP1 in BI-1-deficient cells was shown to further exacerbate the damage caused by the ER stress inducer, tunicamycin (Lisbona et al., 2009). In another *in vitro* study, it was shown that knocking out the *TMBIM6* gene in cells resulted in hyperactivity of the IRE1α pathway, whereas overexpression of BI-1 reversed those effects (Sano and Reed, 2013). The above-mentioned *in vitro* studies provide strong support for BI-1 as a potent inhibitor of ER-stress-induced pathways; however, the exact mechanism by which it may exert its pro-survival signaling remains unknown.

The present study is the first to show the relationship between BI-1 and IRE1α signaling in an *in vivo* neonatal HI model. To determine whether BI-1 confers protection via inhibition of IRE1α, we either silenced BI-1 or upregulated IRE1α to counteract the effects of BI-1 (Fig. 4). Treatment with BI-1 siRNA or an IRE1α CRISPR AP significantly reduced BI-1 expression levels (Fig. 5B) and increased levels of pro-apoptotic markers (Fig. 5C–F, Fig. 6B–D) in rat ipsilateral hemispheric tissue samples. By contrast, administration of Ad-TMBIM6 significantly increased BI-1 levels, attenuated pIRE1α, XBP1 and CHOP expression, and reduced pro-apoptotic marker levels via inhibition of IRE1α (Fig. 5, Fig. 6).

The ER dictates the fate of proteins, while the level of redox mediators modulates the level of ROS (Zeeshan et al., 2016; Cao and Kaufman, 2014). Persistent ER stress causes accumulation of misfolded proteins, which triggers IRE1α activation and further upregulation of CHOP. Increased levels of CHOP further exacerbate cell death signaling through oxidative stress which contributes to ROS generation (Zeeshan et al., 2016; Cao and Kaufman, 2014). Herein, we used a ROS marker, reactive oxygen species modulator 1 (ROMO1), which is a protein responsible for increasing ROS in cells. Western blotting data showed a significant reduction in ROMO1 levels in the brain after treatment with Ad-TMBIM6 (Fig. 5F). This reduction was shown to be via BI-1 as either BI-1 knockdown with siRNA or upregulation of IRE1α with CRISPR AP in the presence of Ad-TMBIM6 reversed the effect.

Although BI-1 has not been shown to interact with PERK, a study by Lee et al. (2007) has reported that PERK may affect the BI-1 signaling pathway. The same study also demonstrated that overexpression of BI-1 decreased eIF2α levels, which is indicative of a possible inhibitory effect on the PERK pathway via unknown mechanisms (Lee et al., 2007). By contrast, a separate study showed conflicting results, where levels of eIF2α were unchanged in BI-1 knockout mice (Bailly-Maitre et al., 2006).

Since BI-1 has been implicated in the inhibition of IRE1α and to some extent of PERK (Robinson et al., 2011; Lee et al., 2007), we employed an *in vitro* OGD model to investigate their involvement. In summary, our *in vitro* findings showed that neither activating nor inhibiting PERK changed the level of BI-1 expression induced through Ad-TMBIM6, whereas manipulation of the stress receptor IRE1α did cause changes in BI-1 expression. In addition, expression of BI-1 by means of Ad-TMBIM6 successfully attenuated pIRE1α expression, while inhibition of BI-1 reversed this effect (Fig. 8C). Based on these findings, we can conclude that despite the inhibitory effect of BI-1 on PERK expression (Fig. 8D), inhibiting PERK does not confer neuroprotection, as evaluated through observation of CC3 levels (Fig. 8E). Taken together, all of the above data advocates that the major BI-1 pathway for neuroprotection after ischemic injury is via inhibition of IRE1α signaling.

The lack of understanding of HI pathology and the timing of the therapeutic window, and a lack of mechanistic studies all hinder the investigation of novel therapeutic approaches for HI injury. Studies have demonstrated a wide array of signaling mechanisms and protective properties of BI-1 after injury (Li et al., 2014; Robinson et al., 2011; Kim et al., 2008; Henke et al., 2011). It has been shown that BI-1 can regulate calcium release, bind to and activate Bcl-2 and inhibit Bax, attenuate UPR proteins activated as a result of ER stress, reduce the production of ROS and increase actin polymerization (Li et al., 2014; Robinson et al., 2011; Kim et al., 2008; Henke et al., 2011). These multimodal properties of BI-1 suggest that it can target a wide array of pathophysiological consequences, making it an ideal candidate as a therapeutic target for HI injury. However, BI-1 signaling mechanisms and the role of BI-1 after HI injury are not well understood, which limits its clinical translatability. Therefore, in this study, we focused on two general concepts: to examine the effects of upregulating BI-1 in the neonatal rat brain, and to investigate the mechanisms by which it confers its protective effects. With this study, we revealed some of the multimodal properties of BI-1, its impact after HI injury and the signaling mechanisms involved, which we hope will pave the way to the establishment of BI-1 as a potential therapeutic target for individuals with HIE and other stroke types with similar pathologies. However, while we have demonstrated that BI-1 can confer protection against the effects of HIE via direct inhibition of IRE1α signaling, this mechanism may not be the primary mode of action in other models of disease. Therefore, future studies need to evaluate potential alternative signaling pathways of BI-1, its role in disease, and its possible effects beyond ER stress mechanisms.

## MATERIALS AND METHODS

### *In vivo* experiments

The Institutional Animal Care and Use Committee of Loma Linda University have approved all protocols. The animals were cared for and all studies conducted were in accordance with the United States Public Health Service Policy on Humane Care and Use of Laboratory Animals. All animal experiments comply with the ARRIVE guidelines and were carried out in accordance with the National Institutes of Health guide for the care and use of laboratory animals. Sprague Dawley rat mothers, with litters of 10–12 pups, were purchased from Harlan Labs (Livermore, CA). The following were applied to ensure robust and unbiased experimental design and analysis of data. (A) Randomization and blinding: random numbers were generated and assigned to each animal and group, then Microsoft Excel was used to randomly assign animals into the various experimental groups. To reduce experimenter bias and achieve unbiased results, the investigators performing the neurological tests and molecular experiments were blinded to animal groups and the interventions that animals received. (B) Control Groups: appropriate control groups were included for each intervention group. Furthermore, for siRNA experiments, animals were injected with only siRNA or scramble siRNA, without the Ad-TMBIM6 vector treatment, as control. No animals were excluded from analysis as long as they survived until the chosen euthanasia times.

### Hypoxic ischemic rat model

HI surgery was performed using the established Rice Vannucci model as previously described (Vannucci and Vannucci, 1997; Vannucci and Hagberg, 2004). Briefly, postnatal day (P)10 unsexed Sprague Dawley rat pups were placed into a temperature-controlled chamber for induction of general anesthesia. The animals were anesthetized with 3% isoflurane gas in air and maintained at 2.5% isoflurane during surgery. Throughout the surgical and post-operative period, temperature was controlled with heating blankets and incubators. After induction of anesthesia, the neck was prepared and draped following standard sterile techniques. Following this, an incision was made on the anterior neck with a No. 11 blade surgical knife (approximately 3–5 mm in length). Using gentle blunt dissection, the right carotid artery was isolated and gently separated from surrounding structures. The carotid artery was then ligated with 5-0 surgical suture, and the skin closed with sutures. Rats were then allowed to recover for 1 h on a heated blanket. Thereafter, they were placed in a 500-ml airtight jar partially submerged in a 37°C water bath to maintain a constant temperature. The neonatal pups were exposed to a gas mixture of 8% oxygen and 92% nitrogen for 90 min. Subsequently, rats were returned to their mothers and monitored daily.

### Treatment administration

Human adenoviral TMBIM6 vector (Ad-TMBIM6) (ADV-225645, Vector Biolabs) was injected intracerebroventricularly (icv) at 2 µl containing 1.6×10^11^ PFU/ml per injection (Bailly-Maitre et al., 2010) at 72 h, 48 h and 24 h pre-HI and 1 h post-HI. BI-1 siRNA (sc-270613, Santa Cruz Biotechnology) and IREα CRISPR activation plasmid (AP) (sc-400576-ACT, Santa Cruz Biotechnology) were administered icv at 48 h pre-HI. Scramble siRNA (sc-37007, Santa Cruz Biotechnology) and control CRISPR (sc-437275, Santa Cruz Biotechnology) were also administered to control animals.

### 2,3,5-triphenyltetrazolium chloride monohydrate staining

Animals were euthanized at 72 h post-HI and infarct area was assessed using 2,3,5-triphenyltetrazolium chloride monohydrate (TTC) staining. Briefly, brains were sectioned into a total of five slices/brain with each slice being 2 mm thick. The slices were then immersed in 2% TTC solution until the color turned red (red is indicative of healthy, living tissue and white shows the infarcted tissue), as previously described (Zhou et al., 2009; Li et al., 2012). Slices were washed from TTC solution using PBS and stored in 10% formaldehyde solution overnight. To calculate the percentage infarcted area of total brain area, ImageJ software was used. For statistical analysis, data was presented as mean±s.d. using one-way ANOVA followed by Tukey multiple-comparison post hoc analysis test, showing significance at *P<0.05 versus sham; ^#^*P*<0.05 versus vehicle; ^@^*P*<0.05 versus Ad-TMBIM6; ^$^*P*<0.05 versus BI-1 siRNA; ^+^*P*<0.05 versus Ad-TMBIM6+scramble siRNA; ^&^*P*<0.05 versus IRE1α CRISPR AP; ^%^*P*<0.05 versus control CRISPR. *n*=6/group.

### Behavioral analysis

Neurological deficits were assessed at four weeks post-HI using water maze and foot fault tests. We chose these neurobehavioral tests because they effectively cover a range of neurological functioning (sensation, motor function and cognitive functioning). In addition, these tests have a good predictive value of impaired neurological function and the water maze test has been closely correlated with long-term functional deficits. Animals were then euthanized, and brain samples weighed and collected for histopathological examination.

#### Foot fault

Animals were placed on a horizontal grid floor (28 squares measuring 3 cm^2^, wire diameter 0.4 cm) for 2 min. Foot fault is defined as when the animal inaccurately places a hind limb, and it falls through one of the openings in the grid. The numbers of foot faults for each hind limb of each animal was recorded (Bouet et al., 2010; Hartman and Warren, 2005).

#### Water maze

Morris water maze test was used to assess learning and memory deficits (Bouet et al., 2010; Hartman and Warren, 2005). The test runs over four days which are also referred to as block 1–4. Each animal performs five trials per day with a 10-min interval between successive trials. Before memory and learning testing begins, each animal is trained using a visible platform (diameter, 10 cm; cued test, block 1). Animals are dropped into the pool of water from four different locations: north-east, north-west, south-east, south-west, and the time taken and distance traveled to find the platform is recorded. Each trial lasts no longer than 1 min. On days 2–4 (blocks 2–4), latency and distance traveled to find a submerged platform (1 cm below the water) was measured (memory blocks 2–4, five trials each block, 1 min each trial). At the start of each new day of memory testing, the location of the submerged platform was changed. The animals’ swim paths were tracked and recorded using a Video Tracking System SMART-2000 (San Diego Instruments Inc.). Animals were then euthanized, and brains collected for histological analysis. For behavior testing, one-way ANOVA and one-way repeated measures of variance followed by Tukey multiple-comparison or Holm–Sidak post hoc analysis test were used showing significance at *P<0.05 versus sham; ^#^*P*<0.05 versus vehicle.

### Western blotting of brain tissue samples

Western blotting was performed as previously described (Li et al., 2015). Once rats were fully anesthetized, they were transcardially perfused with 100 ml ice-cold PBS (pH 7.4). The brain was removed on ice and divided into contralateral and ipsilateral hemispheres, following which it was immediately snap frozen in liquid nitrogen and stored at −80°C. To prepare samples for western blotting, tissue was weighed and the ipsilateral hemispheres were homogenized in RIPA lysis buffer (Santa Cruz Biotechnology) for 15 min followed by centrifugation, 14,000 ***g*** at 4°C for 30 min. The pellet was discarded and supernatant was collected. Protein concentration was measured using a detergent compatibility assay (DC Protein Assay Kit II, Bio-Rad, 5000112). Equal amounts of protein (30 μg) were loaded on an 8–12% SDS-PAGE gel and electrophoresed and then transferred to a nitrocellulose membrane (0.2 μm). Membrane was blocked for 1 h and then incubated with primary antibodies overnight at 4°C: anti-BI-1 (1:100, ab18852, Abcam), anti-IRE1α or anti-pIRE1α (1:100, ab48187 and ab37073, Abcam), anti-XBP1 (1:100, ab37152, Abcam), anti-CHOP (1:500, 15204-1-AP, Proteintech), anti-Casp3 and anti-cleaved caspase 3 (1:400, 9661S and 9662S, Cell Signaling Technology), anti-Bcl2 (1:500, ab194583, Abcam), anti-Bax (1:500, ab32503, Abcam) and anti-ROMO1 (1:100, SAB2107329, Sigma-Aldrich). Membranes were then incubated at room temperature for 1 h with horseradish peroxidase-conjugated secondary antibodies (1:2000, sc-2357, Santa Cruz Biotechnology) followed by chemiluminescence detection (GE Healthcare). The densitometry quantification of the bands was performed with ImageJ software. For statistical analysis of western blot bands, one-way ANOVA followed by Tukey multiple-comparison post hoc analysis was used showing significance at *P<0.05 versus sham; ^#^*P*<0.05 versus vehicle; ^@^*P*<0.05 versus Ad-TMBIM6; ^$^*P*<0.05 versus BI-1 siRNA; ^+^*P*<0.05 versus Ad-TMBIM6+scramble siRNA; ^&^*P*<0.05 versus IRE1α CRISPR AP; ^%^*P*<0.05 versus control CRISPR; ^^^*P*<0.05 versus Ad-TMBIM6+ BI-1 siRNA; ^ε^*P*<0.05 versus BI-1 siRNA-only group. *n*=6/group. Data presented as mean±s.d.

### Histology

Rats were anesthetized at 72 h post-HI and transcardially perfused with 100 ml of PBS and 100 ml of formalin. Brains were then removed and post-fixed by immersion in formalin overnight. The tissue was then dehydrated in 30% sucrose solution for 3–5 days, following which brains were frozen in OCT, and then sectioned into 10 μm thick coronal slices by cryostat (CM3050S; Leica Microsystems) for immunohistochemistry and Fluoro-Jade C staining, and into 16–20 μm thickness for Nissl staining.

#### Immunofluorescence staining

Immunofluorescence staining was performed as previously described (Shi et al., 2017). Tissue was mounted in slides and washed with 0.1 M PBS three times for 5 min, following which it was incubated in 0.3% Triton X-100 in 0.1 M PBS for 30 min at room temperature. Slices were then washed using 0.1 M PBS for 5 min, three times, and the primary antibodies were applied overnight at 4°C: anti-BI-1 (1:100, ab18852, Abcam), anti-IRE1α or anti-pIRE1α (1:100, ab48187 and ab37073, Abcam), anti-NeuN (1:100, ab104224, Abcam), anti-GFAP (1:100, ab53554, Abcam), anti-Iba1 (1:100, ab5076, Abcam), anti-CD11b/c (1:100, ab1211, Abcam) and anti-CC3 (1:100, 9661S, Abcam). After washing with PBS, sections were incubated with the appropriate secondary antibodies: anti-rabbit IgG-TR (711-295-152), anti-mouse IgG-FITC (715-095-150), anti-goat IgG-FITC (705-095-008), anti-rabbit IgG-FITC (711-095-152) (all at 1:200, Jackson ImmunoResearch) for 1 h at room temperature. Finally, slides were covered with DAPI and visualized using a fluorescence microscope. To analyze the images, Magna Fire SP system (Olympus) was used.

#### Fluoro-Jade C staining

Brain sections of 10 μm thickness were cut with a cryostat (Leica LM3050S) for Fluoro-Jade C staining as previously described (Xie et al., 2017). Slides were immersed in 1% sodium hydroxide in 80% ethanol for 5 min, followed by rinsing for 2 min in 70% ethanol, then 2 min in distilled water. Slides were then incubated in 0.06% potassium permanganate solution for 10 min, 2 min in distilled water, and transferred into a 0.0001% solution of Fluoro-Jade C (Millipore), which was dissolved in 0.1% acetic acid. Slides were then rinsed three times with distilled water for 1 min each. Water was drained, and slides dried and finally cleared with xylene for 1 min and a cover slip treated with DPX (Sigma-Aldrich) was placed. We chose Fluoro-Jade C over its predecessors, Fluoro-Jade and Fluoro-Jade B because it has been shown to be the most sensitive and exhibits the highest affinity for degenerating neurons, producing the highest resolution and contrast (Schmued et al., 2005).

#### Nissl staining and evaluation of brain tissue loss and ventricular area

Pups were euthanized at 4 weeks post-HI and brains were sectioned. The prepared slides (16 μm) were dehydrated in 95% and 70% ethanol for 1 min each, rinsed in tap water and then distilled water for 30 s. Slices were then stained with 0.5% Cresyl Violet (Sigma-Aldrich) for 2 min, and washed in distilled water for 10 and 30 s. 100% ethanol and xylene was used to dehydrate for 1.5 min, two times, before a coverslip with Permount was placed (Shi et al., 2017). ImageJ was used to measure percentage tissue loss.

### *In vitro* experiments

Rat pheochromocytoma cells, PC-12 cells, were used at passage 6–9. Cells were grown as previously described (Dickson et al., 1986). Briefly, cells were cultured in full growth media which was composed of FK12 media enriched with 15% horse serum, 2.5% FBS and 5 ml of penicillin. Cells were kept in an incubator at 37°C receiving 5% CO_2_, replacing media every 3 days until they reached 70% confluence. For western blotting, cells were plated at a density of 20,000 cells/well in a 6-well plate.

Primary neuronal cells were collected from rat pups at postnatal day (P)1, as previously described (https://www.jove.com/science-education/5214/primary-neuronal-cultures). Rats were anesthetized and their brain cortices were collected and kept in cold HBSS solution. The cortices were carefully dissected and stored in trypsin-EDTA for 15 min to digest tissue. Following incubation, the tissue was washed with FK12 media with 15% FBS to remove trypsin and stop the enzymatic reaction. The remaining tissue was further softened by titration, following which cells were plated on poly D-lysine-coated flasks and placed in an incubator at 37°C and 5% CO_2_. Once plated, the neurons were left to grow inside a humidified incubator and media was replaced every 3 days until 80% confluence was reached. The cells were then ready to passage and use for analysis. For western blotting, cells were plated at a density of 20,000 cells/well in a 6-well plate.

### Oxygen glucose deprivation model

Cell culture medium of PC-12 cells was replaced with glucose-deprived medium (pure FK12 medium without being enriched with horse serum, FBS or penicillin), after which cells were placed in a hypoxic chamber and flushed with 1% oxygen for 1 h, 1.5 h, 3 h, 5 h and 6 h. Complete growth media was then added and cells allowed to recover for 18 h, after which the cells were prepared for cell death assay and western blotting (Souvenir et al., 2014; Agani et al., 2002).

### Cell death assay

Trypan exclusion was used to determine cell death as previously described (Souvenir et al., 2014). Briefly, cells were scraped and then centrifuged for 5 min, after which they were re-suspended in 10 ml of complete growth medium. A 20 µl aliquot of cells was added to 20 µl Trypan Blue, mixed well, and then let sit for 3 min. 10 µl of this mixture was placed on cell counter slides and the slides were read using an automated cell counter. The mean result of six counts was used for analysis (Souvenir et al., 2011).

### Western blotting for cells

PC-12 cells were collected 18 h after OGD treatment and stored for western blotting as previously described (Souvenir et al., 2014; Dominguez et al., 2008). Briefly, cells were centrifuged [14,000 rpm (20,800 ***g***), 5 min] and then re-suspended in 1 ml PBS, after which they were centrifuged one more time at 14,000 rpm (20,800 ***g***) for 5 min. Supernatant was removed, leaving the pellet. RIPA buffer with protease inhibitor was added to the pellet and pipetted thoroughly until the pellet was fully broken down. The mixture was then allowed to sit on ice for 20 min before being centrifuged at 14,000 rpm (20,800 ***g***) for 30 min. The supernatant was collected while the pellet was discarded. Protein concentration in the supernatant was measured using the Bradford assay and SD-PAGE electrophoresis was performed as previously described (Dominguez et al., 2008). The primary antibodies were: BI-1 antibody (1:100, ab18852, Abcam), anti-IRE1α or anti-pIRE1α antibody (1:100, ab48187 and ab37073, Abcam), anti-PERK antibody (1:100, ab229912, Abcam) and Casp3 and cleaved caspase 3 antibodies (1:400, 9661S and 9662S, Cell Signaling Technology). For statistical analysis, one-way ANOVA followed by Tukey multiple-comparison post hoc analysis was used with significance at *P<0.05 versus control; ^#^*P*<0.05 versus vehicle; ^@^*P*<0.05 versus Ad-TMBIM6 and scramble siRNA or versus MOI=100; ^ε^*P*<0.05 versus Ad-TMBIM6 only; ^$^*P*<0.05 versus BI-1 siRNA; ^&^*P*<0.05 versus APY-29; ^+^*P*<0.05 versus DMSO; ^%^*P*<0.05 versus CCT020312; ^^^*P*<0.05 versus STF-083010 and control CRISPR. *n*=5-6/group.

### Calculation of multiplicity of infection for Ad-TMBIM6

To determine the total amount of infectious particles needed to infect one cell, multiplicity of infection (MOI), was calculated as follows: number of cells×desired MOI=total plaque forming units (PFU) needed; (total PFU needed)/(PFU/ml)=total ml of virus needed to reach your desired dose.

### Cell culture treatments

#### siRNA transfection

Prior to transfection, PC-12 cells were differentiated and allowed to reach 80% confluence in poly D-lysine-coated 6-well plates. Scramble siRNA (sc-37007, Santa Cruz Biotechnology) and siRNA targeting BI-1 (sc-270613, Santa Cruz Biotechnology) was prepared according to manufacturer's protocol. Briefly, a stock of 10 µM siRNA was prepared and 4 µl of this stock was mixed with 125 µl Opti-MEM. In a separate tube, 7 µl Lipofectamine3000 was mixed with 125 µl Opti-MEM. The two tubes were combined and left to sit at room temperature for 15–45 min. 250 µl mixture/well was applied to cells and an additional 1 ml Opti-MEM was added. Cells were then placed in an incubator for 5–7 h, following which transfection medium was removed and replaced with normal growth medium.

#### CRISPR sequence transfection

Prior to transfection, 50,000 PC-12 cells/well were plated on 6-well plates and allowed to grow to 80% confluence. The PC-12 cells were transfected with the desired MOI of Ad-TMBIM6 in 700 µl opti-MEM media and incubated overnight. After 48 h, 700 µl CRISPR plasmid targeting IRE1α (sc-400576-ACT) or control sequence plasmid (sc-437275) (both from Santa Cruz Biotechnology) were added. The plasmids were diluted in 200 µl sterile DNase free water (provided) giving a concentration of 0.1 µg/µl. The stock solution was further diluted with Opti-MEM transfection medium to bring to the desired concentration of 2–8 µg per 0.5–1 million cells. Cells were transfected with CRISPR using Ultracruz transfection reagent. Next, transfected cells were placed in an incubator at 37°C for 5–7 h, after which the medium was removed and replaced with normal medium, and cells prepared for OGD.

#### Treatment with pharmaceutical agonists and antagonists

STF-083010 (IRE1α antagonist, 4509, Tocris), CCT020312 (PERK agonist, 324879, Sigma-Aldrich) and APY-29 (IRE1α agonist, 4865, Tocris) were dissolved and prepared according to manufacturer's recommendations. CCT020312 was used at 10 μM. STF-083010 and APY-29 were tested at final concentrations of 20 μM or 60 μM. The best dose was determined to be 20 μM for both drugs (cell viability quantification data not shown).

### Statistical analysis

Data were analyzed using GraphPad Prism software for statistical analysis. Statistical differences among more than two groups were analyzed using a one-way analysis of variance (ANOVA) followed by Tukey's multiple comparisons test or Holm–Sidak or Dunnett's post hoc analysis. A *P*-value <0.05 was considered significant. Long-term neurobehavioral data were analyzed using Sigma Plot 10.0 and Sigma Stat version 3.5 (Systat Software) using one-way ANOVA. Data is presented as mean±s.d. *P*-values of <0.05 were considered statistically significant. Sample sizes were determined using previous publications from our lab as well as a formal sample size calculation. All sample sizes were calculated assuming a type I error (false positive) rate=0.05 and power of 0.8. Based on previous studies, expected mean values and variation within groups, as well as the expected change in the means (change of 20–50% for western blotting and 30% for long-term neurobehavioral analysis), we concluded that a sample size of 4–6 pups/group for molecular techniques (i.e. western blotting) and 7–9 pups/group for behavior studies were needed. Please refer to Tables S1–S10 for detailed statistical analysis.

## Supplementary Material

Supplementary information
