# Supplementary material for: Adenoviral TMBIM6 vector attenuates ER-stress-induced apoptosis in a neonatal hypoxic-ischemic rat model
Source: Dis Model Mech. 2019 Nov 22;12(11):dmm040352. doi: 10.1242/dmm.040352 (PMC6898997; doi:10.1242/dmm.040352)
Supplement: Supplementary information [file dmm-12-040352-s1.pdf]

Table S1 Statistical Analysis for Endogenous Proteins Expression Levels post-HI

Statistical Data Analysis for Fig. 1B

	Tukey's multiple comparisons test	95% CI of diff.	Significant?	Summary	Adjusted P Value	ANOVA table	DF	F (DFn, DFd)	P value
BI-1 Time Course Western Blot						Treatment (between columns)	4	F (4, 15) = 5.845	P = 0.0049
	Sham vs. 6h	-0.3716 to 0.2680	No	ns	0.9861	Residual (within columns)	15		
	Sham vs. 12h	-0.5935 to 0.04614	No	ns	0.1119	Total	19		
	Sham vs. 24h	-0.7165 to -0.07689	Yes	*	0.0121				
	Sham vs. 72h	-0.3391 to 0.3005	No	ns	0.9997				
	6h vs. 12h	-0.5417 to 0.09790	No	ns	0.2533				
	6h vs. 24h	-0.6647 to -0.02513	Yes	*	0.0317				
	6h vs. 72h	-0.2873 to 0.3523	No	ns	0.9976				
	12h vs. 24h	-0.4428 to 0.1968	No	ns	0.758				
	12h vs. 72h	-0.06540 to 0.5742	No	ns	0.1537				
	24h vs. 72h	0.05763 to 0.6972	Yes	*	0.0173				

Statistical Data Analysis for Fig. 1C

	Tukey's multiple comparisons test	95% CI of diff.	Significant?	Summary	Adjusted P Value	ANOVA table	DF	F (DFn, DFd)	P value
IRE1 Time Course Western Blot						Treatment (between columns)	4	F (4, 15) = 19.35	P < 0.0001
	Sham vs. 6h	-1.567 to -0.7368	Yes	****	< 0.0001	Residual (within columns)	15		
	Sham vs. 12h	-1.214 to -0.3841	Yes	***	0.0002	Total	19		
	Sham vs. 24h	-1.017 to -0.1876	Yes	**	0.0034				
	Sham vs. 72h	-1.038 to -0.2081	Yes	**	0.0025				
	6h vs. 12h	-0.06208 to 0.7676	No	ns	0.1152				
	6h vs. 24h	0.1344 to 0.9640	Yes	**	0.0073				
	6h vs. 72h	0.1139 to 0.9436	Yes	**	0.0099				
	12h vs. 24h	-0.2184 to 0.6113	No	ns	0.6				
	12h vs. 72h	-0.2388 to 0.5908	No	ns	0.6894				
	24h vs. 72h	-0.4353 to 0.3944	No	ns	0.9999				

Statistical Data Analysis for Fig. 1D

	Tukey's multiple comparisons test	95% CI of diff.	Significant?	Summary	Adjusted P Value	ANOVA table	DF	F (DFn, DFd)	P value
XBP1 Timecourse Western Blot						Treatment (between columns)	4	F (4, 15) = 8.499	P = 0.0009
	Sham vs. 6h	-2.098 to -0.3184	Yes	**	0.006	Residual (within columns)	15		
	Sham vs. 12h	-2.232 to -0.4526	Yes	**	0.0024	Total	19		
	Sham vs. 24h	-2.288 to -0.5089	Yes	**	0.0017				
	Sham vs. 72h	-2.217 to -0.4374	Yes	**	0.0027				
	6h vs. 12h	-1.024 to 0.7556	No	ns	0.9894				
	6h vs. 24h	-1.080 to 0.6993	No	ns	0.9618				
	6h vs. 72h	-1.009 to 0.7708	No	ns	0.9932				
	12h vs. 24h	-0.9461 to 0.8334	No	ns	0.9996				
	12h vs. 72h	-0.8746 to 0.9049	No	ns	> 0.9999				
	24h vs. 72h	-0.8183 to 0.9612	No	ns	0.9991				

Statistical Data Analysis for Fig. 1E

	Tukey's multiple comparisons test	95% CI of diff.	Significant?	Summary	Adjusted P Value	ANOVA table	DF	F (DFn, DFd)	P value
CHOP Time Course Western Blot						Treatment (between columns)	4	F (4, 15) = 3.182	P = 0.0443
	Sham vs. 6h	-1.692 to 0.5472	No	ns	0.5318	Residual (within columns)	15		
	Sham vs. 12h	-1.483 to 0.7562	No	ns	0.8504	Total	19		
	Sham vs. 24h	-2.321 to -0.08202	Yes	*	0.0327				
	Sham vs. 72h	-1.960 to 0.2790	No	ns	0.1926				
	6h vs. 12h	-0.9105 to 1.329	No	ns	0.9766				
	6h vs. 24h	-1.749 to 0.4903	No	ns	0.4432				
	6h vs. 72h	-1.388 to 0.8513	No	ns	0.9435				
	12h vs. 24h	-1.958 to 0.2813	No	ns	0.1946				
	12h vs. 72h	-1.597 to 0.6423	No	ns	0.6858				
	24h vs. 72h	-0.7585 to 1.481	No	ns	0.8532				

A summary excel spreadsheet showing detailed statistical analysis tests with exact p values, degrees of freedom and F values for endogenous expression levels of BI-1, pIRE1α, XBP1 and CHOP. Data expressed as mean +/- SD; Test used was one-way ANOVA followed by Tukey multiple-comparison post hoc analysis (n=4)

Table S2 Statistical Analysis for Optimal Time of Ad-TMBIM6 Injection at 72h post-HI

Statistical Data Analysis for Fig. 2A								
Best Time of Vector Delivery	**Vehicle vs Ad-TMBIM6 (72h pre HI)**		**Vehicle vs Ad-TMBIM6 (48h pre HI)**		**Vehicle vs Ad-TMBIM6 (24h pre HI)**		**Vehicle vs Ad-TMBIM6 (1h post HI)**	
	**Unpaired t test**		**Unpaired t test**		**Unpaired t test**		**Unpaired t test**	
	P value	0.1371	P value	0.0024	P value	0.0789	P value	0.5876
	P value summary	ns	P value summary	**	P value summary	ns	P value summary	ns
	Significantly different? (P < 0.05)	No	Significantly different? (P < 0.05)	Yes	Significantly different? (P < 0.05)	No	Significantly different? (P < 0.05)	No
	One- or two-tailed P value?	Two-tailed	One- or two-tailed P value?	Two-tailed	One- or two-tailed P value?	Two-tailed	One- or two-tailed P value?	Two-tailed
	t, df	t=1.715 df=6	t, df	t=4.173 df=9	t, df	t=1.981 df=9	t, df	t=0.5623 df=9
	Difference between means	-5.640 ± 3.288	Difference between means	-9.048 ± 2.168	Difference between means	-5.168 ± 2.609	Difference between means	-1.920 ± 3.414
	95% confidence interval	-13.69 to 2.405	95% confidence interval	-13.95 to -4.144	95% confidence interval	-11.07 to 0.7332	95% confidence interval	-9.642 to 5.803

A summary excel spreadsheet showing detailed statistical analysis tests with exact p values, degrees of freedom and F values for percent infarcted area after administration of Ad-TMBIM6 vector to determine optimal time point of injection. Data expressed as mean +/- SD; Test used was Unpaired T-Test (n=6)

Table S3 Statistical Analysis for Long-term Behavioral Outcomes at 4 weeks post-HI

Statistical Data Analysis for Fig. 3A - Foot fault

	Tukey's multiple comparisons test	95% CI of diff.	Significant?	Summary	Adjusted P Value		ANOVA table	DF	F (DFn, DFd)	P value
Right Foot Fault							Treatment (between columns)	2	F (2, 18) = 13.19	P = 0.0003
	Sham vs. Vehicle	-15.61 to -5.227	Yes	***	0.0002		Residual (within columns)	18		
	Sham vs. Ad-TMBIM6	-10.66 to 0.03675	No	ns	0.0518		Total	20		
	Vehicle vs. Ad-TMBIM6	0.1337 to 10.08	Yes	*	0.0436					

	Tukey's multiple comparisons test	95% CI of diff.	Significant?	Summary	Adjusted P Value		ANOVA table	DF	F (DFn, DFd)	P value
Left Foot Fault							Treatment (between columns)	2	F (2, 19) = 1.327	P = 0.2888
	Sham vs. Vehicle	-6.899 to 1.788	No	ns	0.3156		Residual (within columns)	19		
	Sham vs. Ad-TMBIM6	-5.156 to 4.013	No	ns	0.9464		Total	21		
	Vehicle vs. Ad-TMBIM6	-2.169 to 6.137	No	ns	0.4598					

Statistical Data Analysis for Fig. 3B - Water Maze

	Block 4					ANOVA table	DF	F (DFn, DFd)	P value
Distance TRavelled	Holm-Sidak's multiple comparisons test	Significant?	Summary	Adjusted P Value					
						Treatment (between columns)	2	F (2, 18) = 16.91	P < 0.0001
	Sham vs. Vehicle	Yes	****	< 0.0001		Residual (within columns)	18		
	Sham vs. Ad-TMBIM6	Yes	**	0.0082		Total	20		
	Vehicle vs. Ad-TMBIM6	Yes	*	0.04					

	Block 3					ANOVA table	DF	F (DFn, DFd)	P value
Distance Travelled	Holm-Sidak's multiple comparisons test	Significant?	Summary	Adjusted P Value					
						Treatment (between columns)	2	F (2, 19) = 9.318	P = 0.0015
	Sham vs. Vehicle	Yes	**	0.0012		Residual (within columns)	19		
	Sham vs. Ad-TMBIM6	Yes	*	0.0228		Total	21		
	Vehicle vs. Ad-TMBIM6	No	ns	0.1766					

	Block 2					ANOVA table	DF	F (DFn, DFd)	P value
Distance Travelled	Holm-Sidak's multiple comparisons test	Significant?	Summary	Adjusted P Value					
	Sham vs. Vehicle	Yes	****	< 0.0001		Treatment (between columns)	2	F (2, 19) = 17.44	P < 0.0001
	Sham vs. Ad-TMBIM6	Yes	**	0.0022		Residual (within columns)	19		
	Vehicle vs. Ad-TMBIM6	No	ns	0.0738		Total	21		

	Bock 1					ANOVA table	DF	F (DFn, DFd)	P value
Distance Travelled	Holm-Sidak's multiple comparisons test	Significant?	Summary	Adjusted P Value					
						Treatment (between columns)	2	F (2, 19) = 0.9171	P = 0.4167
	Sham vs. Vehicle	No	ns	0.5226		Residual (within columns)	19		
	Sham vs. Ad-TMBIM6	No	ns	0.5226		Total	21		
	Vehicle vs. Ad-TMBIM6	No	ns	0.9121					

Statistical Data Analysis for Fig. 3C

	Tukey's multiple comparisons test	95% CI of diff.	Significant?	Summary	Adjusted P Value		ANOVA table	DF	F (DFn, DFd)	P value
Brain Weight							Treatment (between columns)	2	F (2, 17) = 15.33	P = 0.0002
	Sham vs. Vehicle	14.27 to 41.58	Yes	***	0.0002		Residual (within columns)	17		
	Sham vs. Ad-TMBIM6	-7.143 to 22.05	No	ns	0.409		Total	19		
	Vehicle vs. Ad-TMBIM6	-34.13 to -6.820	Yes	**	0.0035					

Statistical Data Analysis for Fig. 3D

	Tukey's multiple comparisons test	95% CI of diff.	Significant?	Summary	Adjusted P Value		ANOVA table	DF	F (DFn, DFd)	P value
Tissue Loss							Treatment (between columns)	2	F (2, 18) = 92.53	P < 0.0001
	Sham vs. Vehicle	-47.01 to -31.72	Yes	****	< 0.0001		Residual (within columns)	18		
	Sham vs. Ad-TMBIM6	-21.33 to -5.573	Yes	**	0.0011		Total	20		
	Vehicle vs. Ad-TMBIM6	18.59 to 33.24	Yes	****	< 0.0001					

A summary excel spreadsheet showing detailed statistical analysis tests with exact p values, degrees of freedom and F values for foot fault, watermaze, brain weight measurements and tissue loss calculations. Data expressed as mean +/- SD; Test used was one-way ANOVA followed by Tukey multiple-comparison/or Holm-Sidak post hoc analysis (n=6-8)

Table S4 Statistical Analysis showing the Effects of BI-1 on Percent Infarcted Area at 72h post-HI

Statistical Data Analysis for Fig. 4B									
	**Tukey's multiple comparisons test**	**95% CI of diff.**	**Significant?**	**Summary**	**Adjusted P Value**	**ANOVA table**	**DF**	**F (DFn, DFd)**	**P value**
Percent Infarct Area for all intervention groups						Treatment (between columns)	8	F (8, 49) = 81.85	P < 0.0001
	Sham vs. Vehicle	-41.39 to -30.18	Yes	****	< 0.0001	Residual (within columns)	49		
	Sham vs. Ad-TMBIM6	-23.74 to -12.53	Yes	****	< 0.0001	Total	57		
	Sham vs. BI-1 siRNA	-37.91 to -25.92	Yes	****	< 0.0001				
	Sham vs. Scramble siRNA	-26.65 to -14.67	Yes	****	< 0.0001				
	Sham vs. IRE1 CRISPR (AP)	-36.17 to -24.19	Yes	****	< 0.0001				
	Sham vs. Control CRISPR	-26.66 to -14.68	Yes	****	< 0.0001				
	Sham vs. BI-1 siRNA	-41.04 to -29.06	Yes	****	< 0.0001				
	Sham vs. Scramble siRNA	-38.78 to -26.80	Yes	****	< 0.0001				
	Vehicle vs. Ad-TMBIM6	12.46 to 22.84	Yes	****	< 0.0001				
	Vehicle vs. BI-1 siRNA	-1.735 to 9.473	No	ns	0.3976				
	Vehicle vs. Scramble siRNA	9.521 to 20.73	Yes	****	< 0.0001				
	Vehicle vs. IRE1 CRISPR (AP)	-0.003741 to 11.20	No	ns	0.0503				
	Vehicle vs. Control CRISPR	9.513 to 20.72	Yes	****	< 0.0001				
	Vehicle vs. BI-1 siRNA	-4.870 to 6.338	No	ns	> 0.9999				
	Vehicle vs. Scramble siRNA	-2.609 to 8.600	No	ns	0.723				
	Ad-TMBIM6 vs. BI-1 siRNA	-19.38 to -8.175	Yes	****	< 0.0001				
	Ad-TMBIM6 vs. Scramble siRNA	-8.126 to 3.082	No	ns	0.868				
	Ad-TMBIM6 vs. IRE1 CRISPR (AP)	-17.65 to -6.443	Yes	****	< 0.0001				
	Ad-TMBIM6 vs. Control CRISPR	-8.135 to 3.074	No	ns	0.8659				
	Ad-TMBIM6 vs. BI-1 siRNA	-22.52 to -11.31	Yes	****	< 0.0001				
	Ad-TMBIM6 vs. Scramble siRNA	-20.26 to -9.048	Yes	****	< 0.0001				
	BI-1 siRNA vs. Scramble siRNA	5.266 to 17.25	Yes	****	< 0.0001				
	BI-1 siRNA vs. IRE1 CRISPR (AP)	-4.259 to 7.723	No	ns	0.9895				
	BI-1 siRNA vs. Control CRISPR	5.257 to 17.24	Yes	****	< 0.0001				
	BI-1 siRNA vs. BI-1 siRNA	-9.126 to 2.856	No	ns	0.745				
	BI-1 siRNA vs. Scramble siRNA	-6.864 to 5.118	No	ns	> 0.9999				
	Scramble siRNA vs. IRE1 CRISPR (AP)	-15.52 to -3.534	Yes	***	0.0001				
	Scramble siRNA vs. Control CRISPR	-5.999 to 5.983	No	ns	> 0.9999				
	Scramble siRNA vs. BI-1 siRNA	-20.38 to -8.401	Yes	****	< 0.0001				
	Scramble siRNA vs. Scramble siRNA	-18.12 to -6.139	Yes	****	< 0.0001				
	IRE1 CRISPR (AP) vs. Control CRISPR	3.526 to 15.51	Yes	***	0.0001				
	IRE1 CRISPR (AP) vs. BI-1 siRNA	-10.86 to 1.124	No	ns	0.1989				
	IRE1 CRISPR (AP) vs. Scramble siRNA	-8.596 to 3.386	No	ns	0.8883				
	Control CRISPR vs. BI-1 siRNA	-20.37 to -8.392	Yes	****	< 0.0001				
	Control CRISPR vs. Scramble siRNA	-18.11 to -6.131	Yes	****	< 0.0001				
	BI-1 siRNA vs. Scramble siRNA	-3.729 to 8.253	No	ns	0.947				

A summary excel spreadsheet showing detailed statistical analysis tests with exact p values, degrees of freedom and F values for percent infarcted area on mechanistic data. Data expressed as mean +/- SD; Test used was one-way ANOVA followed by Tukey multiple-comparison post hoc analysis (n=6)

Disease Models & Mechanisms • Supplementary information

Table S5 Statistical Analysis for Western Blot Data Showing Expression of Pathway Proteins at 72h post-HI

Statistical Data Analysis for Fig. 5B

	Tukey's multiple comparisons test	95% CI of diff.	Significant?	Summary	Adjusted P Value		ANOVA table	DF	F (DFn, DFd)	P value
BI-1 Western Blot							Treatment (between columns)	8	F (8, 44) = 10.12	P < 0.0001
	Sham vs. Vehicle	-0.3491 to 0.1348	No	ns	0.8744		Residual (within columns)	44		
	Sham vs. Ad-TMBIM6	-0.6396 to -0.1557	Yes	****	< 0.0001		Total	52		
	Sham vs. BI-1 siRNA	-0.2937 to 0.1902	No	ns	0.9986					
	Sham vs. Scramble siRNA	-0.6640 to -0.1565	Yes	***	0.0001					
	Sham vs. IRE1 CRISPR (AP)	-0.3382 to 0.1457	No	ns	0.927					
	Sham vs. Control CRISPR	-0.6706 to -0.1867	Yes	****	< 0.0001					
	Sham vs. BI-1 siRNA	-0.3469 to 0.1370	No	ns	0.8863					
	Sham vs. Scramble siRNA	-0.3654 to 0.1185	No	ns	0.7644					
	Vehicle vs. Ad-TMBIM6	-0.5325 to -0.04855	Yes	**	0.0086					
	Vehicle vs. BI-1 siRNA	-0.1866 to 0.2973	No	ns	0.9977					
	Vehicle vs. Scramble siRNA	-0.5569 to -0.04934	Yes	**	0.0091					
	Vehicle vs. IRE1 CRISPR (AP)	-0.2311 to 0.2528	No	ns	> 0.9999					
	Vehicle vs. Control CRISPR	-0.5635 to -0.07955	Yes	**	0.0025					
	Vehicle vs. BI-1 siRNA	-0.2398 to 0.2441	No	ns	> 0.9999					
	Vehicle vs. Scramble siRNA	-0.2583 to 0.2256	No	ns	> 0.9999					
	Ad-TMBIM6 vs. BI-1 siRNA	0.1039 to 0.5878	Yes	***	0.0009					
	Ad-TMBIM6 vs. Scramble siRNA	-0.2664 to 0.2412	No	ns	> 0.9999					
	Ad-TMBIM6 vs. IRE1 CRISPR (AP)	0.05943 to 0.5433	Yes	**	0.0056					
	Ad-TMBIM6 vs. Control CRISPR	-0.2730 to 0.2110	No	ns	> 0.9999					
	Ad-TMBIM6 vs. BI-1 siRNA	0.05073 to 0.5346	Yes	**	0.0079					
	Ad-TMBIM6 vs. Scramble siRNA	0.03221 to 0.5161	Yes	*	0.016					
	BI-1 siRNA vs. Scramble siRNA	-0.6122 to -0.1047	Yes	**	0.0011					
	BI-1 siRNA vs. IRE1 CRISPR (AP)	-0.2864 to 0.1975	No	ns	0.9995					
	BI-1 siRNA vs. Control CRISPR	-0.6188 to -0.1349	Yes	***	0.0002					
	BI-1 siRNA vs. BI-1 siRNA	-0.2951 to 0.1888	No	ns	0.9983					
	BI-1 siRNA vs. Scramble siRNA	-0.3136 to 0.1703	No	ns	0.9871					
	Scramble siRNA vs. IRE1 CRISPR (AP)	0.06022 to 0.5677	Yes	**	0.0061					
	Scramble siRNA vs. Control CRISPR	-0.2722 to 0.2354	No	ns	> 0.9999					
	Scramble siRNA vs. BI-1 siRNA	0.05152 to 0.5590	Yes	**	0.0084					
	Scramble siRNA vs. Scramble siRNA	0.03300 to 0.5405	Yes	*	0.0164					
	IRE1 CRISPR (AP) vs. Control CRISPR	-0.5743 to -0.09043	Yes	**	0.0016					
	IRE1 CRISPR (AP) vs. BI-1 siRNA	-0.2507 to 0.2333	No	ns	> 0.9999					
	IRE1 CRISPR (AP) vs. Scramble siRNA	-0.2692 to 0.2147	No	ns	> 0.9999					
	Control CRISPR vs. BI-1 siRNA	0.08173 to 0.5656	Yes	**	0.0023					
	Control CRISPR vs. Scramble siRNA	0.06321 to 0.5471	Yes	**	0.0048					
	BI-1 siRNA vs. Scramble siRNA	-0.2605 to 0.2234	No	ns	> 0.9999					

Statistical Data Analysis for Fig. 5C

	Tukey's multiple comparisons test	95% CI of diff.	Significant?	Summary	Adjusted P Value		ANOVA table	DF	F (DFn, DFd)	P value
pIRE1 Western Blot							Treatment (between columns)	8	F (8, 45) = 44.45	P < 0.0001
	Sham vs. Vehicle	-1.355 to -0.6996	Yes	****	< 0.0001		Residual (within columns)	45		
	Sham vs. Ad-TMBIM6	-0.3282 to 0.3275	No	ns	> 0.9999		Total	53		
	Sham vs. BI-1 siRNA	-1.361 to -0.7052	Yes	****	< 0.0001					
	Sham vs. Scramble siRNA	-0.4805 to 0.1752	No	ns	0.842					
	Sham vs. IRE1 CRISPR (AP)	-1.266 to -0.6104	Yes	****	< 0.0001					
	Sham vs. Control CRISPR	-0.5831 to 0.07266	No	ns	0.2431					
	Sham vs. BI-1 siRNA	-1.309 to -0.6534	Yes	****	< 0.0001					
	Sham vs. Scramble siRNA	-1.293 to -0.6372	Yes	****	< 0.0001					
	Vehicle vs. Ad-TMBIM6	0.6992 to 1.355	Yes	****	< 0.0001					
	Vehicle vs. BI-1 siRNA	-0.3335 to 0.3222	No	ns	> 0.9999					
	Vehicle vs. Scramble siRNA	0.5470 to 1.203	Yes	****	< 0.0001					
	Vehicle vs. IRE1 CRISPR (AP)	-0.2386 to 0.4171	No	ns	0.9926					
	Vehicle vs. Control CRISPR	0.4444 to 1.100	Yes	****	< 0.0001					
	Vehicle vs. BI-1 siRNA	-0.2816 to 0.3741	No	ns	> 0.9999					
	Vehicle vs. Scramble siRNA	-0.2655 to 0.3902	No	ns	0.9994					
	Ad-TMBIM6 vs. BI-1 siRNA	-1.361 to -0.7048	Yes	****	< 0.0001					
	Ad-TMBIM6 vs. Scramble siRNA	-0.4801 to 0.1756	No	ns	0.8437					
	Ad-TMBIM6 vs. IRE1 CRISPR (AP)	-1.266 to -0.6100	Yes	****	< 0.0001					
	Ad-TMBIM6 vs. Control CRISPR	-0.5827 to 0.07302	No	ns	0.2448					
	Ad-TMBIM6 vs. BI-1 siRNA	-1.309 to -0.6530	Yes	****	< 0.0001					
	Ad-TMBIM6 vs. Scramble siRNA	-1.293 to -0.6369	Yes	****	< 0.0001					
	BI-1 siRNA vs. Scramble siRNA	0.5526 to 1.208	Yes	****	< 0.0001					
	BI-1 siRNA vs. IRE1 CRISPR (AP)	-0.2330 to 0.4227	No	ns	0.989					
	BI-1 siRNA vs. Control CRISPR	0.4500 to 1.106	Yes	****	< 0.0001					
	BI-1 siRNA vs. BI-1 siRNA	-0.2760 to 0.3797	No	ns	0.9998					
	BI-1 siRNA vs. Scramble siRNA	-0.2599 to 0.3958	No	ns	0.9989					
	Scramble siRNA vs. IRE1 CRISPR (AP)	-1.113 to -0.4577	Yes	****	< 0.0001					
	Scramble siRNA vs. Control CRISPR	-0.4304 to 0.2253	No	ns	0.982					
	Scramble siRNA vs. BI-1 siRNA	-1.156 to -0.5007	Yes	****	< 0.0001					
	Scramble siRNA vs. Scramble siRNA	-1.140 to -0.4846	Yes	****	< 0.0001					
	IRE1 CRISPR (AP) vs. Control CRISPR	0.3552 to 1.011	Yes	****	< 0.0001					
	IRE1 CRISPR (AP) vs. BI-1 siRNA	-0.3709 to 0.2848	No	ns	> 0.9999					
	IRE1 CRISPR (AP) vs. Scramble siRNA	-0.3547 to 0.3010	No	ns	> 0.9999					
	Control CRISPR vs. BI-1 siRNA	-1.054 to -0.3982	Yes	****	< 0.0001					
	Control CRISPR vs. Scramble siRNA	-1.038 to -0.3820	Yes	****	< 0.0001					
	BI-1 siRNA vs. Scramble siRNA	-0.3117 to 0.3440	No	ns	> 0.9999					

Disease Models & Mechanisms • Supplementary information

Statistical Data Analysis for Fig. 5D

XBP1 Western Blot

Tukey's multiple comparisons test	95% CI of diff.	Significant?	Summary	Adjusted P Value		ANOVA table	DF	F (DFn, DFd)	P value
						Treatment (between columns)	8	F (8, 45) = 32.24	P < 0.0001
Sham vs. Vehicle	-1.602 to -0.8492	Yes	****	< 0.0001		Residual (within columns)	45		
Sham vs. Ad-TMBIM6	-0.5843 to 0.1687	No	ns	0.6833		Total	53		
Sham vs. BI-1 siRNA	-1.226 to -0.4731	Yes	****	< 0.0001					
Sham vs. Scramble siRNA	-0.8010 to -0.04798	Yes	*	0.0168					
Sham vs. IRE1 CRISPR (AP)	-1.383 to -0.6296	Yes	****	< 0.0001					
Sham vs. Control CRISPR	-0.6559 to 0.09719	No	ns	0.3004					
Sham vs. BI-1 siRNA	-1.646 to -0.8932	Yes	****	< 0.0001					
Sham vs. Scramble siRNA	-1.233 to -0.4803	Yes	****	< 0.0001					
Vehicle vs. Ad-TMBIM6	0.6414 to 1.394	Yes	****	< 0.0001					
Vehicle vs. BI-1 siRNA	-0.0004880 to 0.7526	No	ns	0.0505					
Vehicle vs. Scramble siRNA	0.4247 to 1.178	Yes	****	< 0.0001					
Vehicle vs. IRE1 CRISPR (AP)	-0.1570 to 0.5961	No	ns	0.6178					
Vehicle vs. Control CRISPR	0.5698 to 1.323	Yes	****	< 0.0001					
Vehicle vs. BI-1 siRNA	-0.4205 to 0.3325	No	ns	> 0.9999					
Vehicle vs. Scramble siRNA	-0.007655 to 0.7454	No	ns	0.0589					
Ad-TMBIM6 vs. BI-1 siRNA	-1.018 to -0.2653	Yes	****	< 0.0001					
Ad-TMBIM6 vs. Scramble siRNA	-0.5932 to 0.1598	No	ns	0.6339					
Ad-TMBIM6 vs. IRE1 CRISPR (AP)	-1.175 to -0.4218	Yes	****	< 0.0001					
Ad-TMBIM6 vs. Control CRISPR	-0.4480 to 0.3050	No	ns	0.9994					
Ad-TMBIM6 vs. BI-1 siRNA	-1.438 to -0.6854	Yes	****	< 0.0001					
Ad-TMBIM6 vs. Scramble siRNA	-1.026 to -0.2725	Yes	****	< 0.0001					
BI-1 siRNA vs. Scramble siRNA	0.04865 to 0.8017	Yes	*	0.0165					
BI-1 siRNA vs. IRE1 CRISPR (AP)	-0.5330 to 0.2200	No	ns	0.9088					
BI-1 siRNA vs. Control CRISPR	0.1938 to 0.9469	Yes	***	0.0004					
BI-1 siRNA vs. BI-1 siRNA	-0.7966 to -0.04353	Yes	*	0.0186					
BI-1 siRNA vs. Scramble siRNA	-0.3837 to 0.3694	No	ns	> 0.9999					
Scramble siRNA vs. IRE1 CRISPR (AP)	-0.9582 to -0.2051	Yes	***	0.0003					
Scramble siRNA vs. Control CRISPR	-0.2314 to 0.5217	No	ns	0.9387					
Scramble siRNA vs. BI-1 siRNA	-1.222 to -0.4687	Yes	****	< 0.0001					
Scramble siRNA vs. Scramble siRNA	-0.8089 to -0.05581	Yes	*	0.0139					
IRE1 CRISPR (AP) vs. Control CRISPR	0.3503 to 1.103	Yes	****	< 0.0001					
IRE1 CRISPR (AP) vs. BI-1 siRNA	-0.6401 to 0.1130	No	ns	0.3753					
IRE1 CRISPR (AP) vs. Scramble siRNA	-0.2272 to 0.5259	No	ns	0.9286					
Control CRISPR vs. BI-1 siRNA	-1.367 to -0.6139	Yes	****	< 0.0001					
Control CRISPR vs. Scramble siRNA	-0.9540 to -0.2010	Yes	***	0.0003					
BI-1 siRNA vs. Scramble siRNA	0.03636 to 0.7894	Yes	*	0.022					

Statistical Data Analysis for Fig. 5E

CHOP Western Blot

Tukey's multiple comparisons test	95% CI of diff.	Significant?	Summary	Adjusted P Value		ANOVA table	DF	F (DFn, DFd)	P value
						Treatment (between columns)	8	F (8, 45) = 12.56	P < 0.0001
Sham vs. Vehicle	-0.5863 to -0.2146	Yes	****	< 0.0001		Residual (within columns)	45		
Sham vs. Ad-TMBIM6	-0.2791 to 0.09250	No	ns	0.7803		Total	53		
Sham vs. BI-1 siRNA	-0.5347 to -0.1631	Yes	****	< 0.0001					
Sham vs. Scramble siRNA	-0.3436 to 0.02802	No	ns	0.1546					
Sham vs. IRE1 CRISPR (AP)	-0.5427 to -0.1710	Yes	****	< 0.0001					
Sham vs. Control CRISPR	-0.3051 to 0.06652	No	ns	0.4919					
Sham vs. BI-1 siRNA	-0.5139 to -0.1423	Yes	****	< 0.0001					
Sham vs. Scramble siRNA	-0.4918 to -0.1202	Yes	****	< 0.0001					
Vehicle vs. Ad-TMBIM6	0.1213 to 0.4930	Yes	****	< 0.0001					
Vehicle vs. BI-1 siRNA	-0.1342 to 0.2374	No	ns	0.9916					
Vehicle vs. Scramble siRNA	0.05687 to 0.4285	Yes	**	0.0031					
Vehicle vs. IRE1 CRISPR (AP)	-0.1422 to 0.2294	No	ns	0.9973					
Vehicle vs. Control CRISPR	0.09537 to 0.4670	Yes	***	0.0004					
Vehicle vs. BI-1 siRNA	-0.1134 to 0.2582	No	ns	0.9352					
Vehicle vs. Scramble siRNA	-0.09132 to 0.2803	No	ns	0.7687					
Ad-TMBIM6 vs. BI-1 siRNA	-0.4414 to -0.06977	Yes	**	0.0015					
Ad-TMBIM6 vs. Scramble siRNA	-0.2503 to 0.1213	No	ns	0.9662					
Ad-TMBIM6 vs. IRE1 CRISPR (AP)	-0.4494 to -0.07775	Yes	***	0.001					
Ad-TMBIM6 vs. Control CRISPR	-0.2118 to 0.1598	No	ns	> 0.9999					
Ad-TMBIM6 vs. BI-1 siRNA	-0.4206 to -0.04898	Yes	**	0.0047					
Ad-TMBIM6 vs. Scramble siRNA	-0.3985 to -0.02687	Yes	*	0.0143					
BI-1 siRNA vs. Scramble siRNA	0.005282 to 0.3769	Yes	*	0.0396					
BI-1 siRNA vs. IRE1 CRISPR (AP)	-0.1938 to 0.1778	No	ns	> 0.9999					
BI-1 siRNA vs. Control CRISPR	0.04378 to 0.4154	Yes	**	0.0061					
BI-1 siRNA vs. BI-1 siRNA	-0.1650 to 0.2066	No	ns	> 0.9999					
BI-1 siRNA vs. Scramble siRNA	-0.1429 to 0.2287	No	ns	0.9976					
Scramble siRNA vs. IRE1 CRISPR (AP)	-0.3849 to -0.01327	Yes	*	0.0275					
Scramble siRNA vs. Control CRISPR	-0.1473 to 0.2243	No	ns	0.9989					
Scramble siRNA vs. BI-1 siRNA	-0.3561 to 0.01550	No	ns	0.0957					
Scramble siRNA vs. Scramble siRNA	-0.3340 to 0.03762	No	ns	0.2163					
IRE1 CRISPR (AP) vs. Control CRISPR	0.05177 to 0.4234	Yes	**	0.004					
IRE1 CRISPR (AP) vs. BI-1 siRNA	-0.1570 to 0.2146	No	ns	0.9999					
IRE1 CRISPR (AP) vs. Scramble siRNA	-0.1349 to 0.2367	No	ns	0.9923					
Control CRISPR vs. BI-1 siRNA	-0.3946 to -0.02300	Yes	*	0.0173					
Control CRISPR vs. Scramble siRNA	0.3725 to -0.0008819	Yes	*	0.0481					
BI-1 siRNA vs. Scramble siRNA	-0.1637 to 0.2079	No	ns	> 0.9999					

Statistical Data Analysis for Fig. 5F										
ROMO1 Western Blot	Tukey's multiple comparisons test	95% CI of diff.	Significant?	Summary	Adjusted P Value		ANOVA table	DF	F (DFn, DFd)	P value
							Treatment (between columns)	8	F (8, 45) = 15.15	P < 0.0001
	Sham vs. Vehicle	-1.029 to -0.3636	Yes	****	< 0.0001		Residual (within columns)	45		
	Sham vs. Ad-TMBIM6	-0.4161 to 0.2488	No	ns	0.9957		Total	53		
	Sham vs. BI-1 siRNA	-1.016 to -0.3508	Yes	****	< 0.0001					
	Sham vs. Scramble siRNA	-0.6037 to 0.06121	No	ns	0.1926					
	Sham vs. IRE1 CRISPR (AP)	-0.8917 to -0.2268	Yes	****	< 0.0001					
	Sham vs. Control CRISPR	-0.5397 to 0.1252	No	ns	0.5312					
	Sham vs. BI-1 siRNA	-1.019 to -0.3542	Yes	****	< 0.0001					
	Sham vs. Scramble siRNA	-0.9488 to -0.2838	Yes	****	< 0.0001					
	Vehicle vs. Ad-TMBIM6	0.2800 to 0.9449	Yes	****	< 0.0001					
	Vehicle vs. BI-1 siRNA	-0.3196 to 0.3453	No	ns	> 0.9999					
	Vehicle vs. Scramble siRNA	0.09234 to 0.7573	Yes	**	0.0041					
	Vehicle vs. IRE1 CRISPR (AP)	-0.1957 to 0.4693	No	ns	0.9134					
	Vehicle vs. Control CRISPR	0.1563 to 0.8212	Yes	***	0.0006					
	Vehicle vs. BI-1 siRNA	-0.3231 to 0.3418	No	ns	> 0.9999					
	Vehicle vs. Scramble siRNA	-0.2527 to 0.4122	No	ns	0.9969					
	Ad-TMBIM6 vs. BI-1 siRNA	-0.9321 to -0.2671	Yes	****	< 0.0001					
	Ad-TMBIM6 vs. Scramble siRNA	-0.5201 to 0.1448	No	ns	0.6574					
	Ad-TMBIM6 vs. IRE1 CRISPR (AP)	-0.8081 to -0.1432	Yes	***	0.0009					
	Ad-TMBIM6 vs. Control CRISPR	-0.4561 to 0.2088	No	ns	0.9497					
	Ad-TMBIM6 vs. BI-1 siRNA	-0.9355 to -0.2706	Yes	****	< 0.0001					
	Ad-TMBIM6 vs. Scramble siRNA	-0.8651 to -0.2002	Yes	***	0.0001					
	BI-1 siRNA vs. Scramble siRNA	0.07950 to 0.7444	Yes	**	0.0059					
	BI-1 siRNA vs. IRE1 CRISPR (AP)	-0.2085 to 0.4564	No	ns	0.949					
	BI-1 siRNA vs. Control CRISPR	0.1435 to 0.8084	Yes	***	0.0009					
	BI-1 siRNA vs. BI-1 siRNA	-0.3359 to 0.3290	No	ns	> 0.9999					
	BI-1 siRNA vs. Scramble siRNA	-0.2655 to 0.3994	No	ns	0.9991					
	Scramble siRNA vs. IRE1 CRISPR (AP)	-0.6205 to 0.04446	No	ns	0.1375					
	Scramble siRNA vs. Control CRISPR	-0.2685 to 0.3964	No	ns	0.9993					
	Scramble siRNA vs. BI-1 siRNA	-0.7479 to -0.08295	Yes	**	0.0054					
	Scramble siRNA vs. Scramble siRNA	-0.6775 to -0.01259	Yes	*	0.0366					
	IRE1 CRISPR (AP) vs. Control CRISPR	0.01950 to 0.6844	Yes	*	0.0306					
	IRE1 CRISPR (AP) vs. BI-1 siRNA	-0.4599 to 0.2050	No	ns	0.9407					
	IRE1 CRISPR (AP) vs. Scramble siRNA	-0.3895 to 0.2754	No	ns	0.9997					
	Control CRISPR vs. BI-1 siRNA	-0.8118 to -0.1469	Yes	***	0.0008					
	Control CRISPR vs. Scramble siRNA	-0.7415 to -0.07655	Yes	**	0.0064					
	BI-1 siRNA vs. Scramble siRNA	-0.2621 to 0.4028	No	ns	0.9987					

A summary excel spreadsheet showing detailed statistical analysis tests with exact p values, degrees of freedom and F values for western blot experiments measuring the levels of pathway proteins. Data expressed as mean +/- SD; Test used was one-way ANOVA followed by Tukey multiple-comparison post hoc analysis (n=6)

Disease Models & Mechanisms • Supplementary information

Table S6 Statistical Analysis for Western Blot Data Showing Expression of Apoptotic Markers at 72h post-HI

Statistical Data Analysis for Fig. 6B

	Tukey's multiple comparisons test	95% CI of diff.	Significant?	Summary	Adjusted P Value		ANOVA table	DF	F (DFn, DFd)	P value
Bcl-2 Western Blot							Treatment (between columns)	8	F (8, 45) = 16.56	P < 0.0001
	Sham vs. Vehicle	-0.3033 to 0.4743	No	ns	0.9983		Residual (within columns)	45		
	Sham vs. Ad-TMBIM6	-0.8980 to -0.1204	Yes	**	0.003		Total	53		
	Sham vs. BI-1 siRNA	-0.3368 to 0.4408	No	ns	> 0.9999					
	Sham vs. Scramble siRNA	-1.215 to -0.4374	Yes	****	< 0.0001					
	Sham vs. IRE1 CRISPR (AP)	-0.4976 to 0.2800	No	ns	0.9911					
	Sham vs. Control CRISPR	-0.8116 to -0.03404	Yes	*	0.0239					
	Sham vs. BI-1 siRNA	-0.2472 to 0.5304	No	ns	0.9554					
	Sham vs. Scramble siRNA	-0.2577 to 0.5199	No	ns	0.9716					
	Vehicle vs. Ad-TMBIM6	-0.9835 to -0.2059	Yes	***	0.0003					
	Vehicle vs. BI-1 siRNA	-0.4223 to 0.3553	No	ns	> 0.9999					
	Vehicle vs. Scramble siRNA	-1.300 to -0.5229	Yes	****	< 0.0001					
	Vehicle vs. IRE1 CRISPR (AP)	-0.5831 to 0.1945	No	ns	0.7845					
	Vehicle vs. Control CRISPR	-0.8971 to -0.1195	Yes	**	0.003					
	Vehicle vs. BI-1 siRNA	-0.3327 to 0.4449	No	ns	> 0.9999					
	Vehicle vs. Scramble siRNA	-0.3432 to 0.4344	No	ns	> 0.9999					
	Ad-TMBIM6 vs. BI-1 siRNA	0.1724 to 0.9500	Yes	***	0.0008					
	Ad-TMBIM6 vs. Scramble siRNA	-0.7058 to 0.07179	No	ns	0.1933					
	Ad-TMBIM6 vs. IRE1 CRISPR (AP)	0.01154 to 0.7891	Yes	*	0.0392					
	Ad-TMBIM6 vs. Control CRISPR	-0.3025 to 0.4751	No	ns	0.9982					
	Ad-TMBIM6 vs. BI-1 siRNA	0.2620 to 1.040	Yes	****	< 0.0001					
	Ad-TMBIM6 vs. Scramble siRNA	0.2514 to 1.029	Yes	****	< 0.0001					
	BI-1 siRNA vs. Scramble siRNA	-1.267 to -0.4894	Yes	****	< 0.0001					
	BI-1 siRNA vs. IRE1 CRISPR (AP)	-0.5496 to 0.2280	No	ns	0.911					
	BI-1 siRNA vs. Control CRISPR	-0.8636 to -0.08604	Yes	**	0.007					
	BI-1 siRNA vs. BI-1 siRNA	-0.2992 to 0.4784	No	ns	0.9976					
	BI-1 siRNA vs. Scramble siRNA	-0.3097 to 0.4679	No	ns	0.999					
	Scramble siRNA vs. IRE1 CRISPR (AP)	0.3285 to 1.106	Yes	****	< 0.0001					
	Scramble siRNA vs. Control CRISPR	0.01454 to 0.7921	Yes	*	0.0367					
	Scramble siRNA vs. BI-1 siRNA	0.5790 to 1.357	Yes	****	< 0.0001					
	Scramble siRNA vs. Scramble siRNA	0.5684 to 1.346	Yes	****	< 0.0001					
	IRE1 CRISPR (AP) vs. Control CRISPR	0.1000 to 0.5000	Yes	**	0.003					
	IRE1 CRISPR (AP) vs. BI-1 siRNA	-0.1384 to 0.6392	No	ns	0.4875					
	IRE1 CRISPR (AP) vs. Scramble siRNA	-0.1489 to 0.6287	No	ns	0.5449					
	Control CRISPR vs. BI-1 siRNA	0.1756 to 0.9532	Yes	***	0.0007					
	Control CRISPR vs. Scramble siRNA	0.1651 to 0.9427	Yes	***	0.0009					
	BI-1 siRNA vs. Scramble siRNA	-0.3993 to 0.3783	No	ns	> 0.9999					

Statistical Data Analysis for Fig. 6C

	Holm-Sidak's multiple comparisons test	Significant?	Summary	Adjusted P Value			ANOVA table	DF	F (DFn, DFd)	P value
Bax Western Blot							Treatment (between columns)	6	F (6, 35) = 10.21	P < 0.0001
	Sham vs. Vehicle	Yes	****	< 0.0001			Residual (within columns)	35		
	Sham vs. Ad-TMBIM6	No	ns	0.6122			Total	41		
	Sham vs. BI-1 siRNA	Yes	***	0.0007						
	Sham vs. Scramble siRNA	No	ns	0.6032						
	Sham vs. IRE1 CRISPR (AP)	Yes	***	0.0007						
	Sham vs. Control CRISPR	No	ns	0.9051						
	Vehicle vs. Ad-TMBIM6	Yes	**	0.0045						
	Vehicle vs. BI-1 siRNA	No	ns	0.903						
	Vehicle vs. Scramble siRNA	Yes	**	0.0051						
	Vehicle vs. IRE1 CRISPR (AP)	No	ns	0.903						
	Vehicle vs. Control CRISPR	Yes	***	0.0003						
	Ad-TMBIM6 vs. BI-1 siRNA	Yes	*	0.048						
	Ad-TMBIM6 vs. Scramble siRNA	No	ns	0.9969						
	Ad-TMBIM6 vs. IRE1 CRISPR (AP)	Yes	*	0.047						
	Ad-TMBIM6 vs. Control CRISPR	No	ns	0.903						
	BI-1 siRNA vs. Scramble siRNA	Yes	*	0.0481						
	BI-1 siRNA vs. IRE1 CRISPR (AP)	No	ns	0.9969						
	BI-1 siRNA vs. Control CRISPR	Yes	**	0.0038						
	Scramble siRNA vs. IRE1 CRISPR (AP)	Yes	*	0.048						
	Scramble siRNA vs. Control CRISPR	No	ns	0.903						
	IRE1 CRISPR (AP) vs. Control CRISPR	Yes	**	0.0036						

Comparison of all groups vs BI-1 siRNA and scramble siRNA only groups (Without Ad-TMBIM6)

	Holm-Sidak's multiple comparisons test	Mean Diff.	Significant?	Summary	Adjusted P Value	F	ANOVA table	DF
Bax Western Blot							Treatment (between columns)	2
	Sham vs. BI-1 siRNA	-0.6107	Yes	*	0.0102	7.18	Residual (within columns)	15
	Sham vs. Scramble siRNA	-0.5364	Yes	*	0.0161	7.18	Total	17
	Vehicle vs. BI-1 siRNA	0.1752	No	ns	0.6314	0.8279		
	Vehicle vs. Scramble siRNA	0.2495	No	ns	0.5423	0.8279		
	Ad-TMBIM6 vs. BI-1 siRNA	-0.3835	No	ns	0.1308	2.672		
	Ad-TMBIM6 vs. Scramble siRNA	-0.3092	No	ns	0.1888	2.672		
	BI-1 siRNA vs. BI-1 siRNA	0.04422	No	ns	0.9342	0.1496		
	BI-1 siRNA vs. Scramble siRNA	0.1186	No	ns	0.9342	0.1496		
	Scramble siRNA vs. BI-1 siRNA	-0.3738	No	ns	0.1442	2.531		
	Scramble siRNA vs. Scramble siRNA	-0.2994	No	ns	0.2067	2.531		
	IRE1 CRISPR (AP) vs. BI-1 siRNA	0.04967	No	ns	0.9299	0.1672		
	IRE1 CRISPR (AP) vs. Scramble siRNA	0.124	No	ns	0.9228	0.1672		
	Control CRISPR vs. BI-1 siRNA	-0.5254	Yes	*	0.0338	4.866		
	Control CRISPR vs. Scramble siRNA	-0.451	Yes	*	0.0499	4.861		
	BI-1 siRNA vs. Scramble siRNA	0.07437	No	ns	0.6784	7.18		

Disease Models & Mechanisms • Supplementary information

	Statistical Data Analysis for Fig. 6D									
CC3 Western Blot	Holm-Sidak's multiple comparisons test	Significant?	Summary	Adjusted P Value			ANOVA table	DF	F (DFn, DFd)	P value
							Treatment (between columns)	6	F (6, 35) = 14.87	P < 0.0001
	Sham vs. Vehicle	Yes	****	< 0.0001			Residual (within columns)	35		
	Sham vs. Ad-TMBIM6	No	ns	0.9103			Total	41		
	Sham vs. BI-1 siRNA	Yes	***	0.0005						
	Sham vs. Scramble siRNA	No	ns	0.4918						
	Sham vs. IRE1 CRISPR (AP)	Yes	***	0.0005						
	Sham vs. Control CRISPR	No	ns	0.6166						
	Vehicle vs. Ad-TMBIM6	Yes	****	< 0.0001						
	Vehicle vs. BI-1 siRNA	No	ns	0.0772						
	Vehicle vs. Scramble siRNA	Yes	****	< 0.0001						
	Vehicle vs. IRE1 CRISPR (AP)	No	ns	0.0772						
	Vehicle vs. Control CRISPR	Yes	****	< 0.0001						
	Ad-TMBIM6 vs. BI-1 siRNA	Yes	**	0.0056						
	Ad-TMBIM6 vs. Scramble siRNA	No	ns	0.9103						
	Ad-TMBIM6 vs. IRE1 CRISPR (AP)	Yes	**	0.0056						
	Ad-TMBIM6 vs. Control CRISPR	No	ns	0.9103						
	BI-1 siRNA vs. Scramble siRNA	Yes	*	0.0467						
	BI-1 siRNA vs. IRE1 CRISPR (AP)	No	ns	0.9996						
	BI-1 siRNA vs. Control CRISPR	Yes	*	0.0285						
	Scramble siRNA vs. IRE1 CRISPR (AP)	Yes	*	0.0467						
	Scramble siRNA vs. Control CRISPR	No	ns	0.9613						
	IRE1 CRISPR (AP) vs. Control CRISPR	Yes	*	0.0285						
	Comparison of all groups vs BI-1 siRNA and scramble siRNA only groups (Without Ad-TMBIM6)									
CC3 Western blot	Dunnett's multiple comparisons test	95% CI of diff.	Significant?	Summary	Adjusted P Value					
							ANOVA table	DF	F (DFn, DFd)	P value
	Sham vs. BI-1 siRNA	-1.010 to -0.2422	Yes	***	0.0004		Treatment (between columns)	8	F (8, 45) = 7.726	P < 0.0001
	Sham vs. Scramble siRNA	-0.9584 to -0.1909	Yes	**	0.0011		Residual (within columns)	45		
	Vehicle vs. BI-1 siRNA	-0.2400 to 0.5275	No	ns	0.8653		Total	53		
	Vehicle vs. Scramble siRNA	-0.1888 to 0.5787	No	ns	0.6186					
	Ad-TMBIM6 vs. BI-1 siRNA	-0.9233 to -0.1558	Yes	**	0.0024					
	Ad-TMBIM6 vs. Scramble siRNA	-0.8721 to -0.1045	Yes	**	0.007					
	BI-1 siRNA vs. BI-1 siRNA	-0.5224 to 0.2452	No	ns	0.8843					
	BI-1 siRNA vs. Scramble siRNA	-0.4711 to 0.2964	No	ns	0.9904					
	Scramble siRNA vs. BI-1 siRNA	-0.8332 to -0.06571	Yes	*	0.015					
	Scramble siRNA vs. Scramble siRNA	-0.7820 to -0.01445	Yes	*	0.0388					
	IRE1 CRISPR (AP) vs. BI-1 siRNA only	-0.5224 to 0.2451	No	ns	0.8842					
	IRE1 CRISPR (AP) vs. Scramble siRNA only	-0.4711 to 0.2964	No	ns	0.9904					
	Control CRISPR vs. BI-1 siRNA	-0.8588 to -0.09128	Yes	**	0.0091					
	Control CRISPR vs. Scramble siRNA	-0.8075 to -0.04001	Yes	*	0.0244					
	BI-1 siRNA vs. Scramble siRNA	-0.4711 to 0.2964	No	ns	0.9904					

A summary excel spreadsheet showing detailed statistical analysis tests with exact p values, degrees of freedom and F values for western blot experiments measuring the levels of ER stress induced apoptotic markers. Data expressed as mean +/- SD; Test used was one-way ANOVA followed by Tukey multiple-comparison/Holm-Sidak/Dunnett's post hoc analysis (n=6)

Table S7 Statistical Analysis for *in vitro* Cell Viability Studies after OGD

Statistical Data Analysis for Fig. 7A

	Tukey's multiple comparisons test	95% CI of diff.	Significant?	Summary	Adjusted P Value		ANOVA table	DF	F (DFn, DFd)	P value
Time Under OGD							Treatment (between columns)	5	F (5, 25) = 55.57	P < 0.0001
	Control vs. 1h	-2.132 to 24.94	No	ns	0.1353		Residual (within columns)	25		
	Control vs. 1.5h	-2.685 to 23.13	No	ns	0.1808		Total	30		
	Control vs. 3h	32.48 to 58.30	Yes	****	< 0.0001					
	Control vs. 5h	38.23 to 64.05	Yes	****	< 0.0001					
	Control vs. 6h	39.05 to 75.56	Yes	****	< 0.0001					
	1h vs. 1.5h	-14.72 to 12.35	No	ns	0.9998					
	1h vs. 3h	20.45 to 47.52	Yes	****	< 0.0001					
	1h vs. 5h	26.20 to 53.27	Yes	****	< 0.0001					
	1h vs. 6h	27.20 to 64.60	Yes	****	< 0.0001					
	1.5h vs. 3h	22.26 to 48.07	Yes	****	< 0.0001					
	1.5h vs. 5h	28.01 to 53.82	Yes	****	< 0.0001					
	1.5h vs. 6h	28.83 to 65.34	Yes	****	< 0.0001					
	3h vs. 5h	-7.157 to 18.66	No	ns	0.7421					
	3h vs. 6h	-6.336 to 30.17	No	ns	0.3639					
	5h vs. 6h	-12.09 to 24.42	No	ns	0.8993					

Statistical Data Analysis for Fig. 7B

	Tukey's multiple comparisons test	95% CI of diff.	Significant?	Summary	Adjusted P Value		ANOVA table	DF	F (DFn, DFd)	P value
MOI Test							Treatment (between columns)	5	F (5, 30) = 18.79	P < 0.0001
	Control vs. Vehicle	26.31 to 56.74	Yes	****	< 0.0001		Residual (within columns)	30		
	Control vs. 100 MOI	-13.88 to 16.55	No	ns	0.9998		Total	35		
	Control vs. 200 MOI	5.242 to 35.67	Yes	**	0.0037					
	Control vs. 500 MOI	-3.716 to 26.72	No	ns	0.2259					
	Control vs. 1000 MOI	4.367 to 34.80	Yes	**	0.0058					
	Vehicle vs. 100 MOI	-55.41 to -24.98	Yes	****	< 0.0001					
	Vehicle vs. 200 MOI	-36.29 to -5.854	Yes	**	0.0027					
	Vehicle vs. 500 MOI	-45.24 to -14.81	Yes	****	< 0.0001					
	Vehicle vs. 1000 MOI	-37.16 to -6.729	Yes	**	0.0017					
	100 MOI vs. 200 MOI	3.909 to 34.34	Yes	**	0.0074					
	100 MOI vs. 500 MOI	-5.049 to 25.38	No	ns	0.3488					
	100 MOI vs. 1000 MOI	3.034 to 33.47	Yes	*	0.0116					
	200 MOI vs. 500 MOI	-24.17 to 6.258	No	ns	0.4865					
	200 MOI vs. 1000 MOI	-16.09 to 14.34	No	ns	> 0.9999					
	500 MOI vs. 1000 MOI	-7.133 to 23.30	No	ns	0.595					

Disease Models & Mechanisms • Supplementary information

Statistical Data Analysis for Fig. 7D

	Tukey's multiple comparisons test	95% CI of diff.	Significant?	Summary	Adjusted P Value		ANOVA table	DF	F (DFn, DFd)	P value
Cell Viability							Treatment (between columns)	10	F (10, 69) = 82.47	P < 0.0001
	Control vs. Vehicle	40.61 to 62.60	Yes	****	< 0.0001		Residual (within columns)	69		
	Control vs. Ad-TMBIM6	-8.728 to 13.27	No	ns	0.9998		Total	79		
	Control vs. Scramble siRNA	8.002 to 30.77	Yes	****	< 0.0001					
	Control vs. BI-1 siRNA	34.94 to 55.81	Yes	****	< 0.0001					
	Control vs. DMSO	3.518 to 28.60	Yes	**	0.0029					
	Control vs. APY-29	45.69 to 69.45	Yes	****	< 0.0001					
	Control vs. CCT020312	32.28 to 57.36	Yes	****	< 0.0001					
	Control vs. STF-083010	-2.119 to 19.26	No	ns	0.2356					
	Control vs. Control CRISPR	5.162 to 28.92	Yes	***	0.0005					
	Control vs. PERK CRISPR	41.90 to 63.89	Yes	****	< 0.0001					
	Vehicle vs. Ad-TMBIM6	-60.33 to -38.34	Yes	****	< 0.0001					
	Vehicle vs. Scramble siRNA	-43.60 to -20.83	Yes	****	< 0.0001					
	Vehicle vs. BI-1 siRNA	-16.66 to 4.204	No	ns	0.6578					
	Vehicle vs. DMSO	-48.09 to -23.01	Yes	****	< 0.0001					
	Vehicle vs. APY-29	-5.914 to 17.84	No	ns	0.8444					
	Vehicle vs. CCT020312	-19.32 to 5.760	No	ns	0.7761					
	Vehicle vs. STF-083010	-53.72 to -32.35	Yes	****	< 0.0001					
	Vehicle vs. Control CRISPR	-46.44 to -22.68	Yes	****	< 0.0001					
	Vehicle vs. PERK CRISPR	-9.706 to 12.29	No	ns	> 0.9999					
	Ad-TMBIM6 vs. Scramble siRNA	5.732 to 28.50	Yes	***	0.0002					
	Ad-TMBIM6 vs. BI-1 siRNA	32.67 to 53.54	Yes	****	< 0.0001					
	Ad-TMBIM6 vs. DMSO	1.248 to 26.33	Yes	*	0.0196					
	Ad-TMBIM6 vs. APY-29	43.42 to 67.18	Yes	****	< 0.0001					
	Ad-TMBIM6 vs. CCT020312	30.01 to 55.09	Yes	****	< 0.0001					
	Ad-TMBIM6 vs. STF-083010	-4.389 to 16.99	No	ns	0.6749					
	Ad-TMBIM6 vs. Control CRISPR	2.892 to 26.65	Yes	**	0.0043					
	Ad-TMBIM6 vs. PERK CRISPR	39.63 to 61.62	Yes	****	< 0.0001					
	Scramble siRNA vs. BI-1 siRNA	15.15 to 36.83	Yes	****	< 0.0001					
	Scramble siRNA vs. DMSO	-16.21 to 9.551	No	ns	0.9986					
	Scramble siRNA vs. APY-29	25.95 to 50.42	Yes	****	< 0.0001					
	Scramble siRNA vs. CCT020312	12.56 to 38.32	Yes	****	< 0.0001					
	Scramble siRNA vs. STF-083010	-21.90 to 0.2673	No	ns	0.0619					
	Scramble siRNA vs. Control CRISPR	-14.58 to 9.892	No	ns	0.9999					
	Scramble siRNA vs. PERK CRISPR	22.13 to 44.89	Yes	****	< 0.0001					
	BI-1 siRNA vs. DMSO	-41.36 to -17.27	Yes	****	< 0.0001					
	BI-1 siRNA vs. APY-29	0.8360 to 23.55	Yes	*	0.0252					
	BI-1 siRNA vs. CCT020312	-12.60 to 11.50	No	ns	> 0.9999					
	BI-1 siRNA vs. STF-083010	-46.91 to -26.70	Yes	****	< 0.0001					
	BI-1 siRNA vs. Control CRISPR	-39.69 to -16.97	Yes	****	< 0.0001					
	BI-1 siRNA vs. PERK CRISPR	-2.913 to 17.95	No	ns	0.3821					
	DMSO vs. APY-29	28.19 to 54.83	Yes	****	< 0.0001					
	DMSO vs. CCT020312	14.86 to 42.68	Yes	****	< 0.0001					
	DMSO vs. STF-083010	-19.76 to 4.780	No	ns	0.6278					
	DMSO vs. Control CRISPR	-12.34 to 14.30	No	ns	> 0.9999					
	DMSO vs. PERK CRISPR	24.30 to 49.38	Yes	****	< 0.0001					
	APY-29 vs. CCT020312	-26.06 to 0.5745	No	ns	0.0728					
	APY-29 vs. STF-083010	-60.59 to -37.41	Yes	****	< 0.0001					
	APY-29 vs. Control CRISPR	-53.23 to -27.83	Yes	****	< 0.0001					
	APY-29 vs. PERK CRISPR	-16.55 to 7.205	No	ns	0.964					
	CCT020312 vs. STF-083010	-48.52 to -23.99	Yes	****	< 0.0001					
	CCT020312 vs. Control CRISPR	-41.10 to -14.46	Yes	****	< 0.0001					
	CCT020312 vs. PERK CRISPR	-4.469 to 20.61	No	ns	0.552					
	STF-083010 vs. Control CRISPR	-3.120 to 20.06	No	ns	0.3619					
	STF-083010 vs. PERK CRISPR	33.64 to 55.01	Yes	****	< 0.0001					
	Control CRISPR vs. PERK CRISPR	23.98 to 47.73	Yes	****	< 0.0001					

A summary excel spreadsheet showing detailed statistical analysis tests with exact p values, degrees of freedom and F values for the percent cell viability data. Data expressed as mean +/- SD; Test used was one-way ANOVA followed by Tukey multiple-comparison post hoc analysis (n=6)

Table S8 Statistical Analysis for Protein Expression Levels of Pathway Proteins after OGD

Statistical Data Analysis for Fig. 8B										
	**Tukey's multiple comparisons test**	**95% CI of diff.**	**Significant?**	**Summary**	**Adjusted P Value**		**ANOVA table**	**DF**	**F (DFn, DFd)**	**P value**
							Treatment (between columns)	10	F (10, 54) = 9.784	P < 0.0001
BI-1 Western Blot	Control vs. Vehicle	-0.9614 to 0.5107	No	ns	0.9936		Residual (within columns)	54		
	Control vs. Ad-TMBIM6	-1.752 to -0.2799	Yes	**	0.0011		Total	64		
	Control vs. Scramble siRNA	-1.846 to -0.3735	Yes	***	0.0002					
	Control vs. BI-1 siRNA	-0.6351 to 0.8370	No	ns	> 0.9999					
	Control vs. DMSO	-1.674 to -0.2021	Yes	**	0.0034					
	Control vs. APY-29	-1.038 to 0.4338	No	ns	0.9482					
	Control vs. CCT020312	-1.681 to -0.2090	Yes	**	0.0031					
	Control vs. STF-083010	-1.791 to -0.3190	Yes	***	0.0006					
	Control vs. Control CRISPR	-1.816 to -0.2719	Yes	**	0.0015					
	Control vs. PERK CRISPR	-1.909 to -0.4368	Yes	****	< 0.0001					
	Vehicle vs. Ad-TMBIM6	-1.527 to -0.05455	Yes	*	0.0256					
	Vehicle vs. Scramble siRNA	-1.620 to -0.1481	Yes	**	0.0073					
	Vehicle vs. BI-1 siRNA	-0.4097 to 1.062	No	ns	0.9169					
	Vehicle vs. DMSO	-1.449 to 0.02322	Yes	ns	0.0456					
	Vehicle vs. APY-29	-0.8129 to 0.6592	No	ns	> 0.9999					
	Vehicle vs. CCT020312	-1.456 to 0.01635	No	ns	0.0606					
	Vehicle vs. STF-083010	-1.566 to -0.09363	Yes	*	0.0154					
	Vehicle vs. Control CRISPR	-1.591 to -0.04658	Yes	*	0.029					
	Vehicle vs. PERK CRISPR	-1.683 to -0.2114	Yes	**	0.003					
	Ad-TMBIM6 vs. Scramble siRNA	-0.8296 to 0.6425	No	ns	> 0.9999					
	Ad-TMBIM6 vs. BI-1 siRNA	0.3809 to 1.853	Yes	***	0.0002					
	Ad-TMBIM6 vs. DMSO	-0.6583 to 0.8138	No	ns	> 0.9999					
	Ad-TMBIM6 vs. APY-29	-0.02230 to 1.450	Yes	ns	0.0449					
	Ad-TMBIM6 vs. CCT020312	-0.6651 to 0.8069	No	ns	> 0.9999					
	Ad-TMBIM6 vs. STF-083010	-0.7751 to 0.6970	No	ns	> 0.9999					
	Ad-TMBIM6 vs. Control CRISPR	-0.7999 to 0.7440	No	ns	> 0.9999					
	Ad-TMBIM6 vs. PERK CRISPR	-0.8929 to 0.5792	No	ns	0.9997					
	Scramble siRNA vs. BI-1 siRNA	0.4744 to 1.946	Yes	****	< 0.0001					
	Scramble siRNA vs. DMSO	-0.5647 to 0.9074	No	ns	0.9993					
	Scramble siRNA vs. APY-29	0.07125 to 1.543	Yes	*	0.0206					
	Scramble siRNA vs. CCT020312	-0.5716 to 0.9005	No	ns	0.9995					
	Scramble siRNA vs. STF-083010	-0.6816 to 0.7905	No	ns	> 0.9999					
	Scramble siRNA vs. Control CRISPR	-0.7064 to 0.8376	No	ns	> 0.9999					
	Scramble siRNA vs. PERK CRISPR	-0.7994 to 0.6727	No	ns	> 0.9999					
	BI-1 siRNA vs. DMSO	-1.775 to -0.3031	Yes	***	0.0007					
	BI-1 siRNA vs. APY-29	-1.139 to 0.3329	No	ns	0.751					
	BI-1 siRNA vs. CCT020312	-1.782 to -0.3100	Yes	***	0.0007					
	BI-1 siRNA vs. STF-083010	-1.892 to -0.4200	Yes	***	0.0001					
	BI-1 siRNA vs. Control CRISPR	-1.917 to -0.3729	Yes	***	0.0003					
	BI-1 siRNA vs. PERK CRISPR	-2.010 to -0.5377	Yes	****	< 0.0001					
	DMSO vs. APY-29	-0.1001 to 1.372	No	ns	0.1493					
	DMSO vs. CCT020312	-0.7429 to 0.7292	No	ns	> 0.9999					
	DMSO vs. STF-083010	-0.8529 to 0.6192	No	ns	> 0.9999					
	DMSO vs. Control CRISPR	-0.8777 to 0.6662	No	ns	> 0.9999					
	DMSO vs. PERK CRISPR	-0.9707 to 0.5014	No	ns	0.9913					
	APY-29 vs. CCT020312	-1.379 to 0.09320	No	ns	0.1394					
	APY-29 vs. STF-083010	-1.489 to -0.01678	Yes	*	0.0409					
	APY-29 vs. Control CRISPR	-1.514 to 0.03027	Yes	ns	0.0499					
	APY-29 vs. PERK CRISPR	-1.607 to -0.1346	Yes	**	0.0088					
	CCT020312 vs. STF-083010	-0.8460 to 0.6261	No	ns	> 0.9999					
	CCT020312 vs. Control CRISPR	-0.8708 to 0.6731	No	ns	> 0.9999					
	CCT020312 vs. PERK CRISPR	-0.9638 to 0.5083	No	ns	0.9931					
	STF-083010 vs. Control CRISPR	-0.7608 to 0.7831	No	ns	> 0.9999					
	STF-083010 vs. PERK CRISPR	-0.8538 to 0.6183	No	ns	> 0.9999					
	Control CRISPR vs. PERK CRISPR	-0.9009 to 0.6431	No	ns	> 0.9999					

Disease Models & Mechanisms • Supplementary information

Statistical Data Analysis for Fig. 8C

	Tukey's multiple comparisons test	95% CI of diff.	Significant?	Summary	Adjusted P Value		ANOVA table	DF	F (DFn, DFd)	P value
pIRE1 Western Blot							Treatment (between columns)	10	F (10, 55) = 26.07	P < 0.0001
	Control vs. Vehicle	-1.268 to -0.5391	Yes	****	< 0.0001		Residual (within columns)	55		
	Control vs. Ad-TMBIM6	-0.6357 to 0.09348	No	ns	0.329		Total	65		
	Control vs. Scramble siRNA	-0.6327 to 0.09642	No	ns	0.3443					
	Control vs. BI-1 siRNA	-1.256 to -0.5269	Yes	****	< 0.0001					
	Control vs. DMSO	-0.6661 to 0.06302	No	ns	0.1943					
	Control vs. APY-29	-1.141 to -0.4117	Yes	****	< 0.0001					
	Control vs. CCT020312	-1.297 to -0.5677	Yes	****	< 0.0001					
	Control vs. STF-083010	-0.3155 to 0.4137	No	ns	> 0.9999					
	Control vs. Control CRISPR	-0.5480 to 0.1812	No	ns	0.8346					
	Control vs. PERK CRISPR	-1.266 to -0.5369	Yes	****	< 0.0001					
	Vehicle vs. Ad-TMBIM6	0.2680 to 0.9972	Yes	****	< 0.0001					
	Vehicle vs. Scramble siRNA	0.2710 to 1.000	Yes	****	< 0.0001					
	Vehicle vs. BI-1 siRNA	-0.3524 to 0.3767	No	ns	> 0.9999					
	Vehicle vs. DMSO	0.2376 to 0.9667	Yes	****	< 0.0001					
	Vehicle vs. APY-29	-0.2372 to 0.4919	No	ns	0.9829					
	Vehicle vs. CCT020312	-0.3932 to 0.3359	No	ns	> 0.9999					
	Vehicle vs. STF-083010	0.5882 to 1.317	Yes	****	< 0.0001					
	Vehicle vs. Control CRISPR	0.3557 to 1.085	Yes	****	< 0.0001					
	Vehicle vs. PERK CRISPR	-0.3624 to 0.3667	No	ns	> 0.9999					
	Ad-TMBIM6 vs. Scramble siRNA	-0.3616 to 0.3675	No	ns	> 0.9999					
	Ad-TMBIM6 vs. BI-1 siRNA	-0.9850 to -0.2559	Yes	****	< 0.0001					
	Ad-TMBIM6 vs. DMSO	-0.3950 to 0.3341	No	ns	> 0.9999					
	Ad-TMBIM6 vs. APY-29	-0.8698 to -0.1407	Yes	***	0.001					
	Ad-TMBIM6 vs. CCT020312	-1.026 to -0.2967	Yes	****	< 0.0001					
	Ad-TMBIM6 vs. STF-083010	-0.04436 to 0.6848	No	ns	0.1346					
	Ad-TMBIM6 vs. Control CRISPR	-0.2769 to 0.4523	No	ns	0.9991					
	Ad-TMBIM6 vs. PERK CRISPR	-0.9950 to -0.2659	Yes	****	< 0.0001					
	Scramble siRNA vs. BI-1 siRNA	-0.9879 to -0.2588	Yes	****	< 0.0001					
	Scramble siRNA vs. DMSO	-0.3980 to 0.3312	No	ns	> 0.9999					
	Scramble siRNA vs. APY-29	-0.8727 to -0.1436	Yes	***	0.0009					
	Scramble siRNA vs. CCT020312	-1.029 to -0.2996	Yes	****	< 0.0001					
	Scramble siRNA vs. STF-083010	-0.04729 to 0.6819	No	ns	0.1429					
	Scramble siRNA vs. Control CRISPR	-0.2798 to 0.4493	No	ns	0.9994					
	Scramble siRNA vs. PERK CRISPR	-0.9979 to -0.2688	Yes	****	< 0.0001					
	BI-1 siRNA vs. DMSO	0.2254 to 0.9545	Yes	****	< 0.0001					
	BI-1 siRNA vs. APY-29	-0.2494 to 0.4798	No	ns	0.9919					
	BI-1 siRNA vs. CCT020312	-0.4054 to 0.3238	No	ns	> 0.9999					
	BI-1 siRNA vs. STF-083010	0.5761 to 1.305	Yes	****	< 0.0001					
	BI-1 siRNA vs. Control CRISPR	0.3436 to 1.073	Yes	****	< 0.0001					
	BI-1 siRNA vs. PERK CRISPR	-0.3746 to 0.3546	No	ns	> 0.9999					
	DMSO vs. APY-29	-0.8393 to -0.1102	Yes	**	0.0025					
	DMSO vs. CCT020312	-0.9953 to -0.2662	Yes	****	< 0.0001					
	DMSO vs. STF-083010	-0.01389 to 0.7153	No	ns	0.0693					
	DMSO vs. Control CRISPR	-0.2464 to 0.4827	No	ns	0.9902					
	DMSO vs. PERK CRISPR	-0.9645 to -0.2354	Yes	****	< 0.0001					
	APY-29 vs. CCT020312	-0.5206 to 0.2086	No	ns	0.9333					
	APY-29 vs. STF-083010	0.4609 to 1.190	Yes	****	< 0.0001					
	APY-29 vs. Control CRISPR	0.2284 to 0.9575	Yes	****	< 0.0001					
	APY-29 vs. PERK CRISPR	-0.4898 to 0.2394	No	ns	0.9849					
	CCT020312 vs. STF-083010	0.6169 to 1.346	Yes	****	< 0.0001					
	CCT020312 vs. Control CRISPR	0.3844 to 1.114	Yes	****	< 0.0001					
	CCT020312 vs. PERK CRISPR	-0.3338 to 0.3954	No	ns	> 0.9999					
	STF-083010 vs. Control CRISPR	-0.5971 to 0.1321	No	ns	0.5542					
	STF-083010 vs. PERK CRISPR	-1.315 to -0.5861	Yes	****	< 0.0001					
	Control CRISPR vs. PERK CRISPR	-1.083 to -0.3536	Yes	****	< 0.0001					

Disease Models & Mechanisms • Supplementary information

Statistical Data Analysis for Fig. 8D

	Tukey's multiple comparisons test	95% CI of diff.	Significant?	Summary	Adjusted P Value		ANOVA table	DF	F (DFn, DFd)	P value
pPERK Western Blot							Treatment (between columns)	10	F (10, 44) = 27.90	P < 0.0001
	Control vs. Vehicle	-0.7482 to -0.3560	Yes	****	< 0.0001		Residual (within columns)	44		
	Control vs. Ad-TMBIM6	-0.4093 to -0.01704	Yes	*	0.0232		Total	54		
	Control vs. Scramble siRNA	-0.3836 to 0.008598	No	ns	0.072					
	Control vs. BI-1 siRNA	-0.7691 to -0.3769	Yes	****	< 0.0001					
	Control vs. DMSO	-0.4056 to -0.01334	Yes	*	0.0275					
	Control vs. APY-29	0.4002 to -0.007946	Yes	*	0.0352					
	Control vs. CCT020312	-0.7763 to -0.3841	Yes	****	< 0.0001					
	Control vs. STF-083010	-0.3722 to 0.02003	No	ns	0.1139					
	Control vs. Control CRISPR	-0.3800 to 0.01223	No	ns	0.0836					
	Control vs. PERK CRISPR	-0.1617 to 0.2306	No	ns	> 0.9999					
	Vehicle vs. Ad-TMBIM6	0.1428 to 0.5350	Yes	****	< 0.0001					
	Vehicle vs. Scramble siRNA	0.1684 to 0.5607	Yes	****	< 0.0001					
	Vehicle vs. BI-1 siRNA	-0.2170 to 0.1752	No	ns	> 0.9999					
	Vehicle vs. DMSO	0.1465 to 0.5387	Yes	****	< 0.0001					
	Vehicle vs. APY-29	0.1519 to 0.5441	Yes	****	< 0.0001					
	Vehicle vs. CCT020312	-0.2242 to 0.1680	No	ns	> 0.9999					
	Vehicle vs. STF-083010	0.1799 to 0.5721	Yes	****	< 0.0001					
	Vehicle vs. Control CRISPR	0.1721 to 0.5643	Yes	****	< 0.0001					
	Vehicle vs. PERK CRISPR	0.3904 to 0.7826	Yes	****	< 0.0001					
	Ad-TMBIM6 vs. Scramble siRNA	-0.1705 to 0.2218	No	ns	> 0.9999					
	Ad-TMBIM6 vs. BI-1 siRNA	-0.5560 to -0.1637	Yes	****	< 0.0001					
	Ad-TMBIM6 vs. DMSO	-0.1924 to 0.1998	No	ns	> 0.9999					
	Ad-TMBIM6 vs. APY-29	-0.1870 to 0.2052	No	ns	> 0.9999					
	Ad-TMBIM6 vs. CCT020312	-0.5631 to -0.1709	Yes	****	< 0.0001					
	Ad-TMBIM6 vs. STF-083010	-0.1591 to 0.2332	No	ns	0.9999					
	Ad-TMBIM6 vs. Control CRISPR	-0.1668 to 0.2254	No	ns	> 0.9999					
	Ad-TMBIM6 vs. PERK CRISPR	0.05149 to 0.4437	Yes	**	0.0042					
	Scramble siRNA vs. BI-1 siRNA	-0.5816 to -0.1894	Yes	****	< 0.0001					
	Scramble siRNA vs. DMSO	-0.2181 to 0.1742	No	ns	> 0.9999					
	Scramble siRNA vs. APY-29	-0.2127 to 0.1796	No	ns	> 0.9999					
	Scramble siRNA vs. CCT020312	-0.5888 to -0.1965	Yes	****	< 0.0001					
	Scramble siRNA vs. STF-083010	-0.1847 to 0.2076	No	ns	> 0.9999					
	Scramble siRNA vs. Control CRISPR	-0.1925 to 0.1998	No	ns	> 0.9999					
	Scramble siRNA vs. PERK CRISPR	0.02585 to 0.4181	Yes	*	0.0152					
	BI-1 siRNA vs. DMSO	0.1674 to 0.5597	Yes	****	< 0.0001					
	BI-1 siRNA vs. APY-29	0.1728 to 0.5651	Yes	****	< 0.0001					
	BI-1 siRNA vs. CCT020312	-0.2033 to 0.1889	No	ns	> 0.9999					
	BI-1 siRNA vs. STF-083010	0.2008 to 0.5930	Yes	****	< 0.0001					
	BI-1 siRNA vs. Control CRISPR	0.1930 to 0.5852	Yes	****	< 0.0001					
	BI-1 siRNA vs. PERK CRISPR	0.4113 to 0.8036	Yes	****	< 0.0001					
	DMSO vs. APY-29	-0.1907 to 0.2015	No	ns	> 0.9999					
	DMSO vs. CCT020312	-0.5668 to -0.1746	Yes	****	< 0.0001					
	DMSO vs. STF-083010	-0.1628 to 0.2295	No	ns	> 0.9999					
	DMSO vs. Control CRISPR	-0.1705 to 0.2217	No	ns	> 0.9999					
	DMSO vs. PERK CRISPR	0.04779 to 0.4400	Yes	**	0.0051					
	APY-29 vs. CCT020312	-0.5722 to -0.1800	Yes	****	< 0.0001					
	APY-29 vs. STF-083010	-0.1681 to 0.2241	No	ns	> 0.9999					
	APY-29 vs. Control CRISPR	-0.1759 to 0.2163	No	ns	> 0.9999					
	APY-29 vs. PERK CRISPR	0.04239 to 0.4346	Yes	**	0.0067					
	CCT020312 vs. STF-083010	0.2080 to 0.6002	Yes	****	< 0.0001					
	CCT020312 vs. Control CRISPR	0.2002 to 0.5924	Yes	****	< 0.0001					
	CCT020312 vs. PERK CRISPR	0.4185 to 0.8107	Yes	****	< 0.0001					
	STF-083010 vs. Control CRISPR	-0.2039 to 0.1883	No	ns	> 0.9999					
	STF-083010 vs. PERK CRISPR	0.01442 to 0.4067	Yes	*	0.0262					
	Control CRISPR vs. PERK CRISPR	0.02221 to 0.4145	Yes	*	0.0182					

Statistical Data Analysis for Fig. 8E

	Tukey's multiple comparisons test	95% CI of diff.	Significant?	Summary	Adjusted P Value	ANOVA table	DF	F (DFn, DFd)	P value
CC3 Western Blot						Treatment (between columns)	10	F (10, 44) = 22.61	P < 0.0001
	Control vs. Vehicle	-1.323 to -0.6641	Yes	****	< 0.0001	Residual (within columns)	44		
	Control vs. Ad-TMBIM6	-0.6322 to 0.02655	No	ns	0.0965	Total	54		
	Control vs. Scramble siRNA	-0.5948 to 0.06396	No	ns	0.2182				
	Control vs. BI-1 siRNA	-1.142 to -0.4837	Yes	****	< 0.0001				
	Control vs. DMSO	-0.6936 to -0.03477	Yes	*	0.0195				
	Control vs. APY-29	-1.131 to -0.4723	Yes	****	< 0.0001				
	Control vs. CCT020312	-0.7080 to -0.04921	Yes	*	0.0129				
	Control vs. STF-083010	-0.3456 to 0.3132	No	ns	> 0.9999				
	Control vs. Control CRISPR	-0.5849 to 0.07388	No	ns	0.2641				
	Control vs. PERK CRISPR	-0.5739 to 0.08486	No	ns	0.3219				
	Vehicle vs. Ad-TMBIM6	0.3612 to 1.020	Yes	****	< 0.0001				
	Vehicle vs. Scramble siRNA	0.3986 to 1.057	Yes	****	< 0.0001				
	Vehicle vs. BI-1 siRNA	-0.1490 to 0.5098	No	ns	0.7394				
	Vehicle vs. DMSO	0.2999 to 0.9587	Yes	****	< 0.0001				
	Vehicle vs. APY-29	-0.1376 to 0.5212	No	ns	0.6656				
	Vehicle vs. CCT020312	0.2855 to 0.9443	Yes	****	< 0.0001				
	Vehicle vs. STF-083010	0.6479 to 1.307	Yes	****	< 0.0001				
	Vehicle vs. Control CRISPR	0.4086 to 1.067	Yes	****	< 0.0001				
	Vehicle vs. PERK CRISPR	0.4195 to 1.078	Yes	****	< 0.0001				
	Ad-TMBIM6 vs. Scramble siRNA	-0.2920 to 0.3668	No	ns	> 0.9999				
	Ad-TMBIM6 vs. BI-1 siRNA	-0.8396 to -0.1808	Yes	***	0.0002				
	Ad-TMBIM6 vs. DMSO	-0.3907 to 0.2681	No	ns	0.9999				
	Ad-TMBIM6 vs. APY-29	-0.8282 to -0.1694	Yes	***	0.0003				
	Ad-TMBIM6 vs. CCT020312	-0.4052 to 0.2536	No	ns	0.9993				
	Ad-TMBIM6 vs. STF-083010	-0.04277 to 0.6160	No	ns	0.1399				
	Ad-TMBIM6 vs. Control CRISPR	-0.2821 to 0.3767	No	ns	> 0.9999				
	Ad-TMBIM6 vs. PERK CRISPR	-0.2711 to 0.3877	No	ns	> 0.9999				
	Scramble siRNA vs. BI-1 siRNA	-0.8770 to -0.2182	Yes	****	< 0.0001				
	Scramble siRNA vs. DMSO	-0.4281 to 0.2307	No	ns	0.9939				
	Scramble siRNA vs. APY-29	-0.8656 to -0.2068	Yes	****	< 0.0001				
	Scramble siRNA vs. CCT020312	-0.4426 to 0.2162	No	ns	0.9831				
	Scramble siRNA vs. STF-083010	-0.08017 to 0.5786	No	ns	0.2964				
	Scramble siRNA vs. Control CRISPR	-0.3195 to 0.3393	No	ns	> 0.9999				
	Scramble siRNA vs. PERK CRISPR	-0.3085 to 0.3503	No	ns	> 0.9999				
	BI-1 siRNA vs. DMSO	0.1195 to 0.7783	Yes	**	0.0015				
	BI-1 siRNA vs. APY-29	-0.3180 to 0.3408	No	ns	> 0.9999				
	BI-1 siRNA vs. CCT020312	0.1051 to 0.7639	Yes	**	0.0024				
	BI-1 siRNA vs. STF-083010	0.4674 to 1.126	Yes	****	< 0.0001				
	BI-1 siRNA vs. Control CRISPR	0.2281 to 0.8869	Yes	****	< 0.0001				
	BI-1 siRNA vs. PERK CRISPR	0.2391 to 0.8979	Yes	****	< 0.0001				
	DMSO vs. APY-29	-0.7669 to -0.1081	Yes	**	0.0021				
	DMSO vs. CCT020312	-0.3438 to 0.3150	No	ns	> 0.9999				
	DMSO vs. STF-083010	0.01856 to 0.6774	Yes	*	0.0306				
	DMSO vs. Control CRISPR	-0.2207 to 0.4380	No	ns	0.9874				
	DMSO vs. PERK CRISPR	-0.2098 to 0.4490	No	ns	0.9749				
	APY-29 vs. CCT020312	0.09366 to 0.7525	Yes	**	0.0034				
	APY-29 vs. STF-083010	0.4560 to 1.115	Yes	****	< 0.0001				
	APY-29 vs. Control CRISPR	0.2167 to 0.8755	Yes	****	< 0.0001				
	APY-29 vs. PERK CRISPR	0.2277 to 0.8865	Yes	****	< 0.0001				
	CCT020312 vs. STF-083010	0.03299 to 0.6918	Yes	*	0.0205				
	CCT020312 vs. Control CRISPR	-0.2063 to 0.4525	No	ns	0.9695				
	CCT020312 vs. PERK CRISPR	-0.1953 to 0.4635	No	ns	0.9467				
	STF-083010 vs. Control CRISPR	-0.5687 to 0.09009	No	ns	0.3518				
	STF-083010 vs. PERK CRISPR	-0.5577 to 0.1011	No	ns	0.4192				
	Control CRISPR vs. PERK CRISPR	-0.3184 to 0.3404	No	ns	> 0.9999				

A summary excel spreadsheet showing detailed statistical analysis tests with exact p values, degrees of freedom and F values for western blot data showing the expression levels of IRE1α and PERK receptor changes as well as pro-apoptotic markers after OGD. Data expressed as mean +/- SD; Test used was one-way ANOVA followed by Tukey multiple-comparison post hoc analysis (n=6)

Table S9 Statistical Analysis for Percent Infarcted Area

Statistical Analysis for Fig. 3B					
One Way Repeated Measures Analysis of Variance					
Normality test: Shapiro-Wilk - passed (p=0.54)					
Comparison	Diff of Means	t	P	P<0.05	F
1h V vs. -48h T	12.569	4.726	0.002	Yes	6.863
1h V vs. -72h T	13.763	4.589	0.002	Yes	
1h T vs. -48h T	10.088	4.038	0.01	Yes	
-24h V vs. -48h T	10.719	4.031	0.01	Yes	
-24h V vs. -72h T	11.913	3.972	0.011	Yes	
1h T vs. -72h T	11.282	3.948	0.011	Yes	
-48h V vs. -48h T	9.609	3.613	0.025	Yes	
-48h V vs. -72h T	10.803	3.602	0.025	Yes	
1h V vs. -24h T	7.579	2.85	0.15	No	
1h V vs. -72h V	8.123	2.708	0.196	No	
-24h T vs. -72h T	6.183	2.164	0.513	No	
-24h V vs. -24h T	5.729	2.154	0.5	No	
-24h V vs. -72h V	6.273	2.091	0.527	No	
1h T vs. -24h T	5.098	2.041	0.542	No	
-24h T vs. -48h T	4.99	1.998	0.551	No	
1h T vs. -72h V	5.642	1.974	0.542	No	
-72h V vs. -72h T	5.64	1.776	0.663	No	
-48h V vs. -24h T	4.619	1.737	0.66	No	
-48h V vs. -72h V	5.163	1.721	0.636	No	
-72h V vs. -48h T	4.447	1.556	0.717	No	
1h V vs. -48h V	2.96	1.053	0.943	No	
1h V vs. 1h T	2.481	0.933	0.955	No	
1h V vs. -24h V	1.85	0.658	0.987	No	
-48h T vs. -72h T	1.193	0.418	0.997	No	
-24h V vs. -48h V	1.11	0.395	0.991	No	
-24h V vs. 1h T	0.631	0.237	0.994	No	
-24h T vs. -72h V	0.543	0.19	0.978	No	
1h T vs. -48h V	0.479	0.18	0.858	No	

A summary excel spreadsheet showing detailed statistical analysis of one way repeated measures analysis of variance followed by Holm-Sidak post hoc test to show difference of Ad-TMBIM6 treatment between groups and across time points. (-72h/-48h/-24h V represents HI+vehicle group at 72h/48h/24h pre HI; -72h/-48h/-24h T represents HI+Ad-TMBIM6 treatment group at 72h/48h/24h pre HI; 1h V or 1h T represents HI+vehicle or HI+Ad-TMBIM6 treatment group at 1h post HI)

Table S10 Statistical Analysis for Long Term Behavioral Data

Statistical Analysis for Fig. 3B										
One Way Repeated Measures Analysis of Variance										
Normality test: Shapiro-Wilk - passed (p=0.175)										
**Comparison**	**Diff of Means**	**t**	**P**	**P<0.05**	**F**	**Comparison**	**Diff of Means**	**t**	**P**	**P<0.05**
Col 15 vs. Col 13	13677.021	2.777	0.039	Yes	8.8	Col 10 vs. Col 11	7361.065	1.841	0.909	No
Col 14 vs. Col 15	11066.741	2.571	0.028	Yes		Col 6 vs. Col 1	7512.862	1.79	0.926	No
Col 6 vs. Col 13	26377.916	6.284	<0.001	Yes		Col 6 vs. Col 7	7119.008	1.781	0.923	No
Col 10 vs. Col 13	25718.026	6.127	<0.001	Yes		Col 14 vs. Col 11	6594.766	1.65	0.963	No
Col 14 vs. Col 13	24951.726	5.945	<0.001	Yes		Col 10 vs. Col 1	6852.972	1.633	0.963	No
Col 2 vs. Col 13	24524.375	5.843	<0.001	Yes		Col 10 vs. Col 7	6459.118	1.616	0.963	No
Col 6 vs. Col 9	22858.42	5.446	<0.001	Yes		Col 2 vs. Col 11	6167.415	1.543	0.975	No
Col 10 vs. Col 9	22198.53	5.289	<0.001	Yes		Col 14 vs. Col 1	6086.673	1.45	0.986	No
Col 3 vs. Col 13	23463.008	5.241	<0.001	Yes		Col 14 vs. Col 7	5692.818	1.424	0.987	No
Col 6 vs. Col 5	21723.018	5.175	<0.001	Yes		Col 2 vs. Col 1	5659.322	1.348	0.992	No
Col 14 vs. Col 9	21432.231	5.106	<0.001	Yes		Col 2 vs. Col 7	5265.467	1.317	0.993	No
Col 10 vs. Col 5	21063.128	5.018	<0.001	Yes		Col 3 vs. Col 11	5106.048	1.19	0.997	No
Col 2 vs. Col 9	21004.88	5.004	<0.001	Yes		Col 3 vs. Col 1	4597.955	1.027	1	No
Col 14 vs. Col 5	20296.829	4.836	<0.001	Yes		Col 7 vs. Col 15	4582.221	1.025	0.999	No
Col 2 vs. Col 5	19869.478	4.734	<0.001	Yes		Col 5 vs. Col 13	4654.897	1	0.999	No
Col 3 vs. Col 9	19943.513	4.455	0.002	Yes		Col 3 vs. Col 7	4204.1	0.98	0.999	No
Col 7 vs. Col 13	19258.908	4.302	0.003	Yes		Col 1 vs. Col 15	4188.367	0.901	1	No
Col 3 vs. Col 5	18808.111	4.201	0.004	Yes		Col 11 vs. Col 15	3680.274	0.823	1	No
Col 11 vs. Col 13	18356.96	4.101	0.006	Yes		Col 9 vs. Col 13	3519.495	0.756	1	No
Col 1 vs. Col 13	18865.053	4.052	0.007	Yes		Col 6 vs. Col 3	2914.907	0.729	1	No
Col 7 vs. Col 9	15739.412	3.516	0.037	Yes		Col 10 vs. Col 3	2255.018	0.564	1	No
Col 11 vs. Col 9	14837.465	3.314	0.067	No		Col 6 vs. Col 2	1853.54	0.503	1	No
Col 1 vs. Col 9	15345.558	3.296	0.07	No		Col 6 vs. Col 14	1426.189	0.387	1	No
Col 7 vs. Col 5	14604.011	3.262	0.075	No		Col 14 vs. Col 3	1488.718	0.372	1	No
Col 11 vs. Col 5	13702.063	3.061	0.128	No		Col 10 vs. Col 2	1193.65	0.324	1	No
Col 1 vs. Col 5	14210.156	3.052	0.128	No		Col 2 vs. Col 3	1061.367	0.265	1	No
Col 6 vs. Col 15	11701.229	2.792	0.245	No		Col 5 vs. Col 9	1135.402	0.244	1	No
Col 10 vs. Col 15	11041.339	2.634	0.344	No		Col 7 vs. Col 11	901.947	0.21	1	No
Col 15 vs. Col 9	11157.191	2.399	0.522	No		Col 10 vs. Col 14	766.3	0.208	1	No
Col 2 vs. Col 15	9847.689	2.349	0.557	No		Col 6 vs. Col 10	659.89	0.179	1	No
Col 15 vs. Col 5	10021.789	2.155	0.72	No		Col 14 vs. Col 2	427.351	0.116	0.999	No
Col 6 vs. Col 11	8020.955	2.006	0.827	No		Col 1 vs. Col 11	508.093	0.114	0.992	No
Col 3 vs. Col 15	8786.322	1.965	0.846	No		Col 7 vs. Col 1	393.854	0.088	0.93	No

A summary excel spreadsheet showing detailed statistical analysis of one way repeated measures analysis of variance followed by Holm-Sidak post hoc test to show difference of Ad-TMBIM6 treatment between groups and across blocks. (Col 1 represents sham group, block 1; Col 2 represents HI+vehicle group, block 1; Col 3 represents HI+Ad-TMBIM6 treatment group, block 1; Col 5 represents sham group, block 2; Col 6 represents HI+vehicle group, block 2; Col 7 represents HI+Ad-TMBIM6 treatment group, block 2; Col 9 represents sham group, block 3; Col 10 represents HI+vehicle group, block 3; Col 11 represents HI+Ad-TMBIM6 treatment group, block 3; Col 13 represents sham group, block 4; Col 14 represents HI+vehicle group, block 4; Col 15 represents HI+Ad-TMBIM6 treatment group, block 4).

Fig. S1 Co-localization of BI-1 with Microglia at 72h post-HI

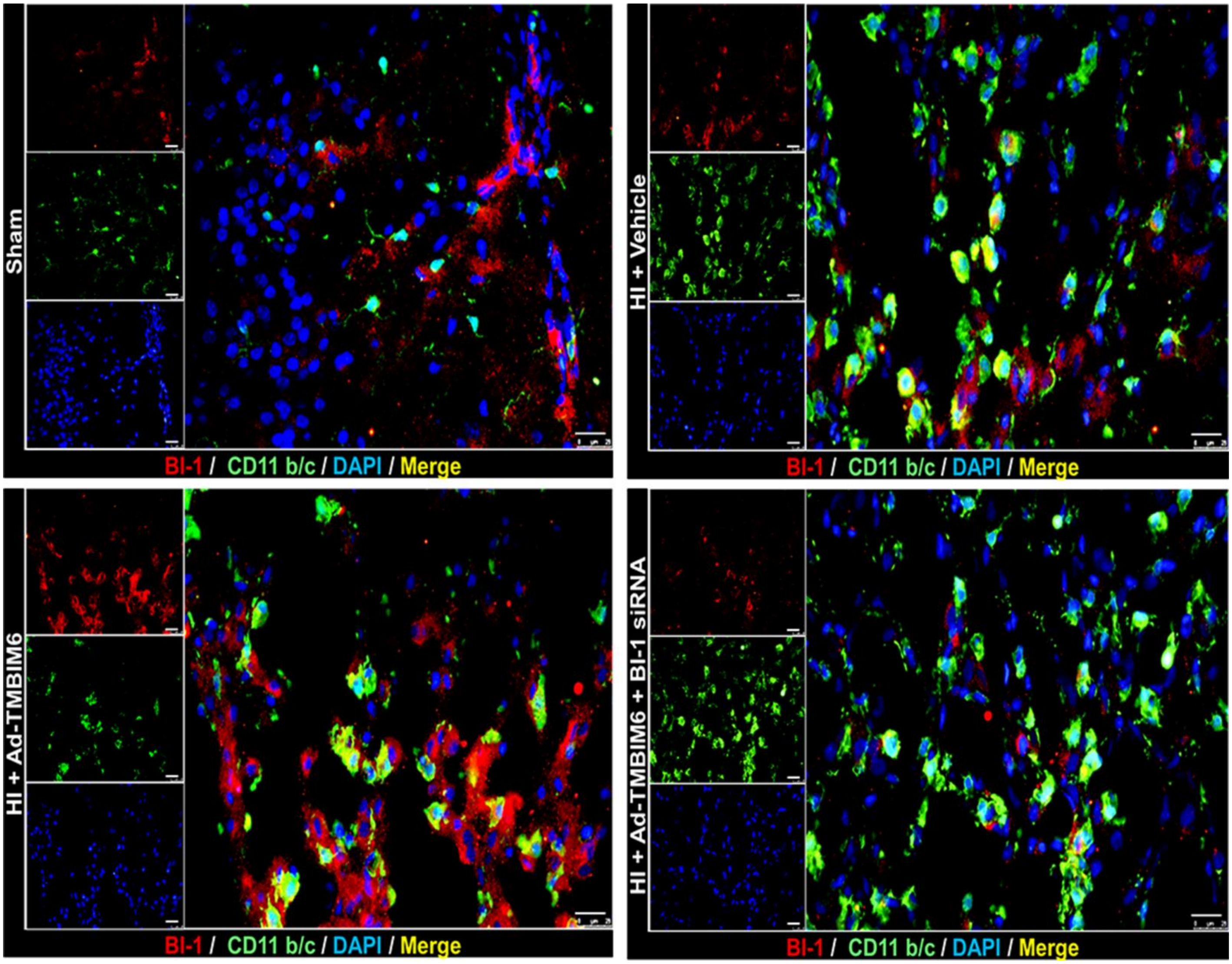

Representative microphotographs of double immunofluorescent staining of BI-1 (red) with microglia (Cd11b/c, green) at 72h post-HI. (Green: microglia; Red: BI-1; Blue: DAPI; Merge shows the co-localization of BI-1 with microglia (yellow); Scale bar = 25µm).

Fig. S2 Expression of pIRE1α with Microglia at per-infarcted area at 72h post-HI

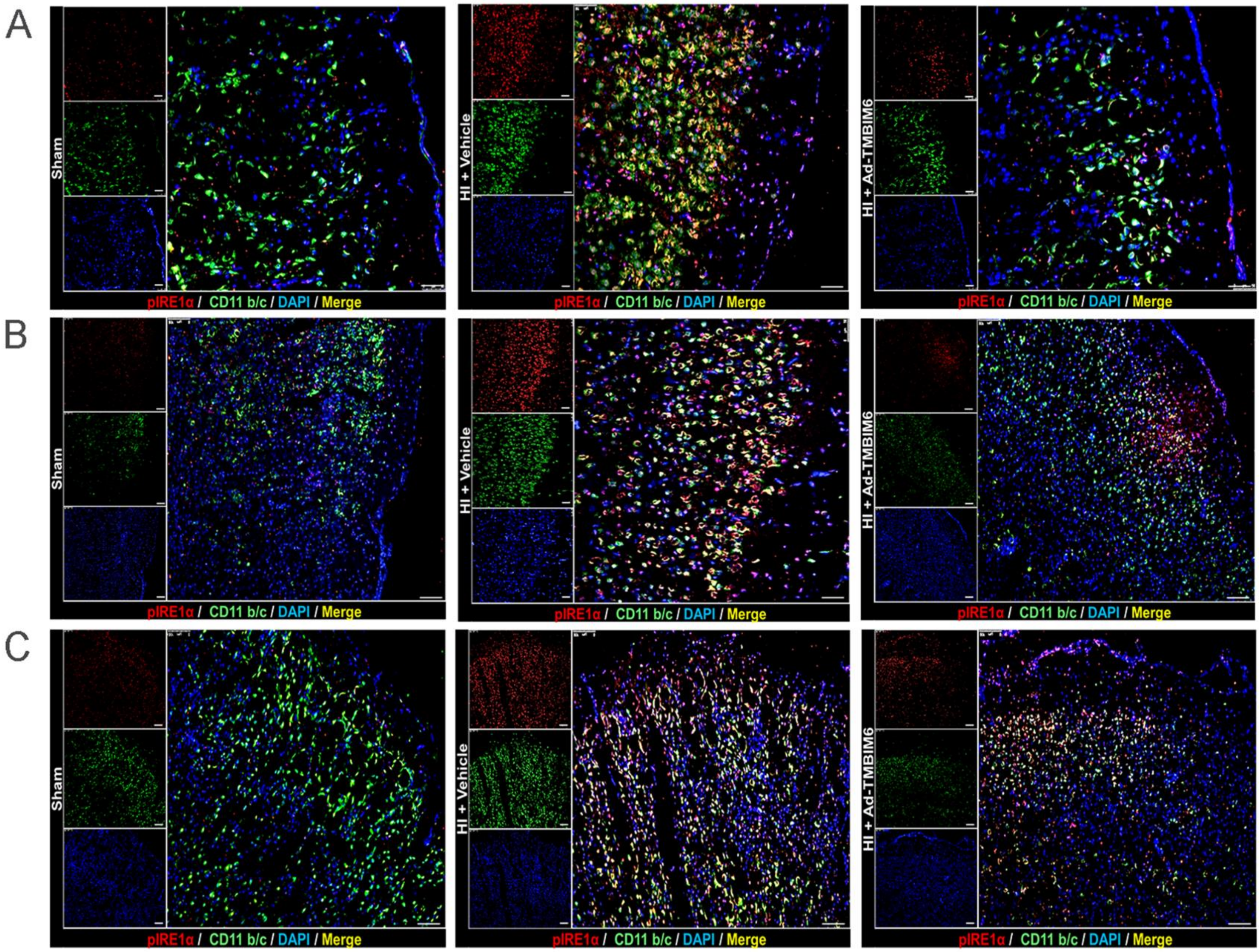

Representative microphotographs of double immunofluorescent staining of pIRE1α (red) with microglia (Cd11b/c, green) at 72h post-HI. Panels A, B and C are images taken from different tissues to show the extent to which the ER stress marker, IRE1α, is spread throughout the injury area. Merge shows the co-localization of BI-1 with microglia (CD11b/c). Scale bar = 100µm

Disease Models & Mechanisms • Supplementary information

Fig. S3 Representative Western Blot bands of key protein expression densities in primary neuronal cell cultures

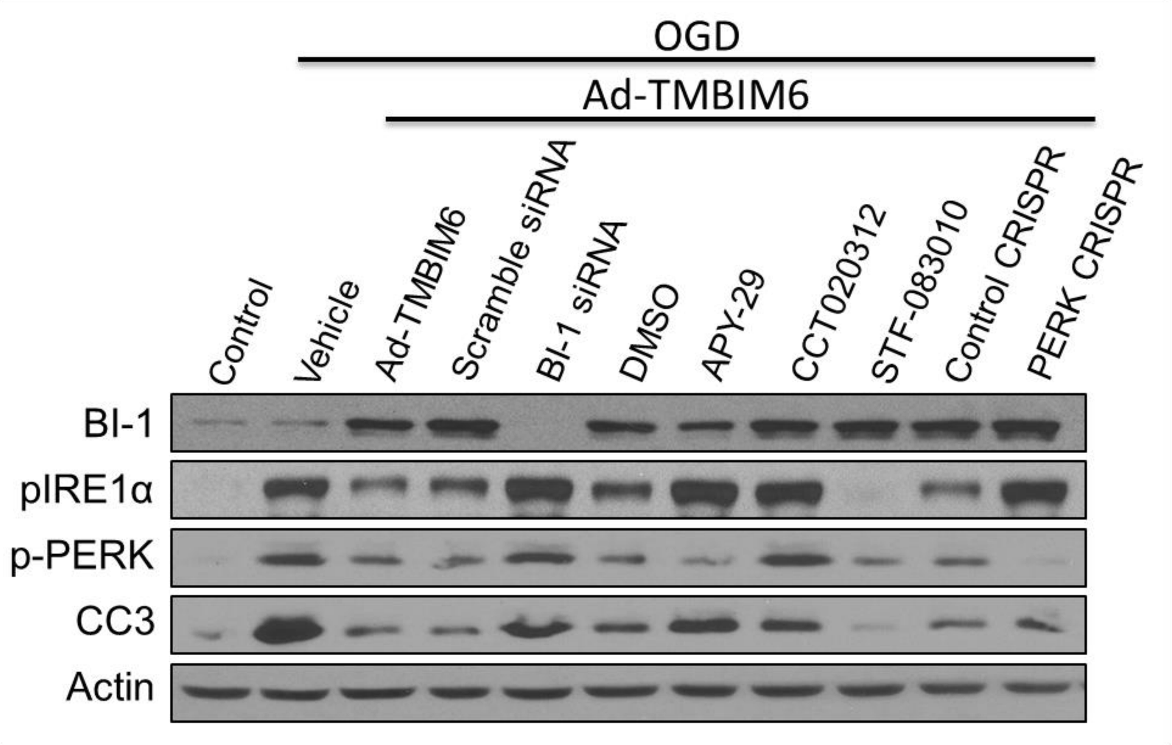

The density of BI-1 protein was higher after Ad-TMBIM6 treatment and in control groups compared to vehicle; while expression densities of pIRE1α, p-PERK and CC3 were reduced after Ad-TMBIM6 treatment in primary neuronal cell cultures.
